# Improving the Hydrophilicity of the Drugs Paclitaxel, Violacein, and Tetrandrine Using Carbon-Based Quantum Dots

**DOI:** 10.3390/nano16181166

**Published:** 2026-09-16

**Authors:** Christine Wu, Katherine Ayala, Solimar Collazo-Hernández, MiaSara Pérez-Salvá, Nataniel Medina-Berríos, Fabiola M. Rosa-Suárez, Alondra Veloz-Bonilla, Rafael A. Villanueva-Nogueras, Alexis Lavín Flores, Keyla A. López-Pérez, Valerie Ortiz-Gómez, Luis Prieto-Costas, Rafael Maldonado-Hernández, Marvin J. Bayro, Gerardo Morell, Brad R. Weiner

**Affiliations:** 1 Department of Chemistry, Smith College, Northampton, MA 01063, USA; wu.christinemass@gmail.com; 2 Department of Chemistry, Berea College, Berea, KY 40404, USA; ayalak@berea.edu; 3 Department of Biology, University of Puerto Rico-Río Piedras Campus, San Juan, PR 00925, USA; solimar.collazo12@gmail.com (S.C.-H.); miasara.perez@upr.edu (M.P.-S.); fabiola.rosa3@upr.edu (F.M.R.-S.); alondra.veloz@upr.edu (A.V.-B.); rafael.villanueva1@upr.edu (R.A.V.-N.); 4 Department of Chemistry, University of Puerto Rico-Río Piedras Campus, San Juan, PR 00925, USA; alexis.lavin@upr.edu (A.L.F.); keyla.lopez1@upr.edu (K.A.L.-P.); marvin.bayro@upr.edu (M.J.B.); 5 National Coalition of Independent Scholars, Brattleboro, VT 05301, USA; 6 Surgical Sciences Department, School of Dental Medicine, University Ana G. Méndez Gurabo Campus, Gurabo, PR 00777, USA; ortizv3@uagm.edu; 7 Center for Tropical Biodiversity, Puerto Rico Science Trust, San Juan, PR 00936, USA; lprieto@prsciencetrust.org; 8 Department of Biology, University of Puerto Rico-Cayey Campus, Cayey, PR 00736, USA; rafael.maldonado1@upr.edu; 9 Department of Physics, University of Puerto Rico-Río Piedras Campus, San Juan, PR 00925, USA; gerardo.morell@upr.edu

**Keywords:** partition coefficient, carbon-based quantum dot, hydrophobic drug, paclitaxel, violacein, tetrandrine

## Abstract

Carbon-based quantum dots and their nitrogen- and sulfur-doped derivatives were used as platforms to improve the aqueous solubility for Paclitaxel, Violacein and Tetrandrine, drugs with known high partition coefficients (Log P). Paclitaxel, Violacein and Tetrandrine showed a significant reduction in their partition coefficient values by coupling with the carbon-based quantum dots. Paclitaxel’s partition coefficient value decreased from 3.96 to 0.2, Violacein’s from 3.34 to 1.17, and Tetrandrine’s from 2.5 to 1.57, thus demonstrating a significant reduction in their hydrophobicity without being encapsulated. The efficiency of each type of carbon-based quantum dot in lowering each drug’s Log P varied and this behavior was correlated with the nature of the surface interaction between each drug and each of the carbon-based quantum dots.

## 1. Introduction

**Figure 1:**
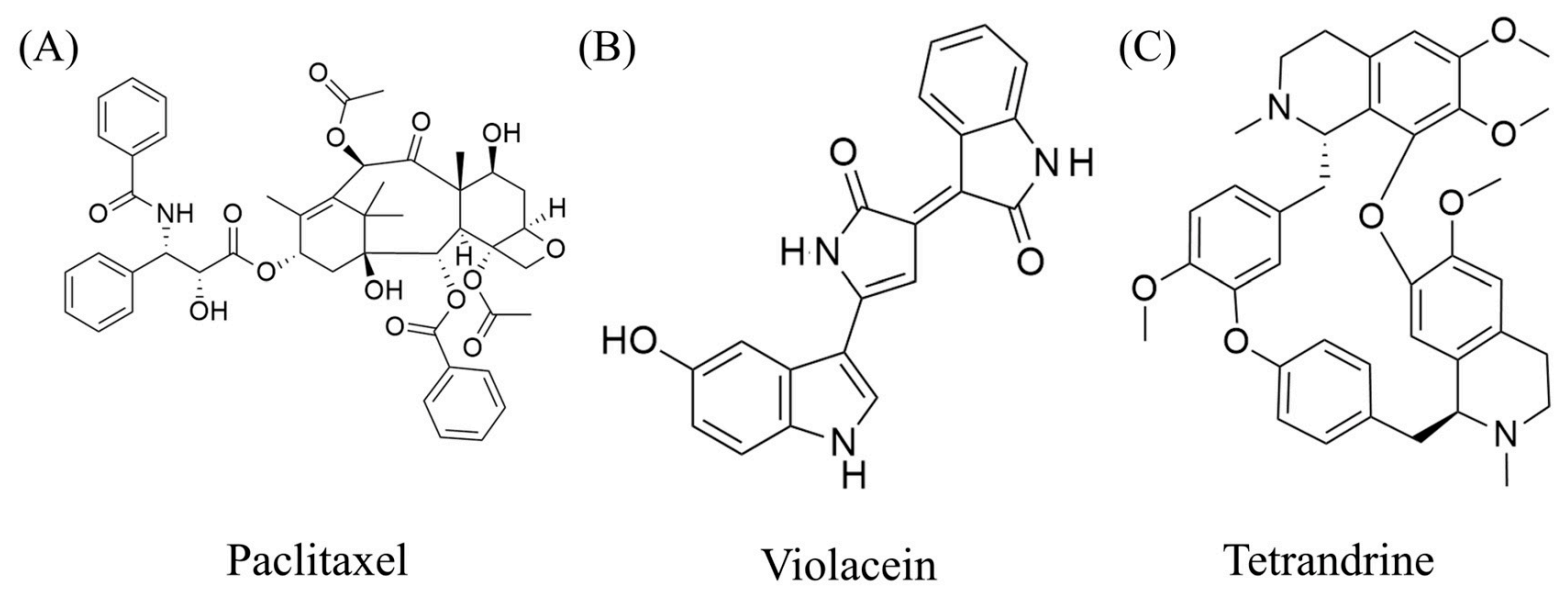
Molecular structures of the hydrophobic drugs used in this study: (**A**) Paclitaxel, (**B**) Violacein and (**C**) Tetrandrine.

The hydrophobicity of drugs can lead to inadequate absorption and distribution throughout the body, which results in lessened effectiveness and rapid clearance in the body [1]. The octanol–water partition coefficient (Log P) describes the equilibrium partitioning of a single, defined charge state of a solute between two liquid phases in contact, such as 1-octanol and water [2]. The measurement of the Log P can define if a solute is hydrophilic, amphiphilic or hydrophobic. Many anti-cancer drugs are hydrophobic, which creates a challenge in physiological uptake and circulation through aqueous phases or tissue fluids [3]. Solubility is related to Log P, but specifically refers to the maximum amount of a substance that can be completely dissolved in a given amount of solvent [4]. Although hydrophobicity helps in crossing the cellular membrane, the low solubility in the aqueous phase can cause aggregation, rapid degradation [5] and local embolisms [3]. Anti-cancer hydrophobic drugs are usually administered intravenously as a systemic delivery that has low targeting efficiency, rapid clearance, and non-specific side effects [6]. The poor water solubility of anti-cancer hydrophobic drugs is still challenging for the controlled delivery and bioavailability of drugs, as they need to be diluted in organic solvents to reach an effective therapeutic dose, resulting in high toxicity [6,7]. In a previous study, we overcame these solubility challenges by employing carbon-based quantum dots (CBQDs) as drug delivery platforms that were able to lower the hydrophobicity of the anti-cancer drug, Andrographolide [8]. CBQDs are zero-dimensional nanoparticles composed of small graphene fragments of around 3–20 nm [9]. CBQDs are known to have chemical stability, photostability and biocompatibility [10] depending on the synthesis method. Adding heteroatoms to the CBQDs to make doped-CBQDs (D-CBQDs) permits tuning of their surface properties [11]. In regards to their capability as drug delivery platforms, one of the known mechanisms of drug loading is the interaction with drugs via their distinctive π-orbitals in the sp^2^-hybridized lattice which can be used to couple drugs through π-π interactions and π-π stacking [12]. Adsorbed drugs are released into the cytoplasm by desorption and diffusion [12]. In the work reported here, we expand the use of the CBQD nanoparticles as platforms for improving the hydrophilicity of the three known hydrophobic drugs with significant anti-cancer activity: Tetrandrine, Violacein and Paclitaxel (see Figure 1).

Tetrandrine is a bisbenzylisoquinoline alkaloid with strong anti-cancer properties despite its low water solubility (0.46 ± 0.11 µg/mL) [13]. It is extracted from the root tuber of the Chinese herb *Stephania tetrandra* S. *Moore.* Tetrandrine has been the focus of several pharmacological studies that indicated the prevention and treatment of multiple cancers, including colon and breast cancer [14,15]. In addition, it has demonstrated many other properties such as anti-microbial [16], anti-depressant [17] and memory-improving activity [18]. The challenge with the usage of the drug is its lack of solubility in water, which leads to poor biodistribution and bioavailability [19].

Violacein, a purple pigment produced by various bacteria of the genus *Chromobacterium*, including *Chromobacterium violaceum*, has emerged as a promising candidate for numerous therapeutic applications due to its diverse biological activities and significant pharmacological potential. Some of these biological activities include antibacterial, antiviral, antitumoral, anti-cancer, antiulcerogenic, and antifungal activities [20,21,22]. However, the clinical translation of Violacein as a drug faces substantial challenges attributed to its low water solubility, i.e., it is reported to have a hydrophobic partition coefficient (Log P) of 3.34 [23], resulting in poor bioavailability and reduced efficacy.

Paclitaxel is a diterpenoid isolated from the bark of the Pacific Yew (*Taxus brevifolia*). It is an effective anticancer drug against a wide range of tumors, such as head and neck, ovarian, breast, and non-small-cell lung cancers [24]. Its low water solubility (0.7 mg/mL) [24] and high hydrophobicity (Log P = 3.96) [25] is still one of the major obstacles for its clinical use [26].

We report here the enhancement of hydrophilicity in Violacein, Tetrandrine, and Paclitaxel through the incorporation of CBQDs, representing a significant advancement in drug delivery systems for these compounds. By improving the water solubility of these hydrophobic drugs, CBQDs can facilitate better bioavailability, reduce side effects, and potentially increase therapeutic efficacy. This development opens the door to new and improved applications, enabling these drugs to be more effectively utilized in clinical settings, particularly in the treatment of diseases where their poor solubility has previously limited their use. Additionally, the use of CBQDs may pave the way for innovative strategies in nanomedicine, enhancing the precision and targeting of drug delivery while minimizing systemic toxicity. For this reason, in this study, we have focused on enhancing the solubility of these hydrophobic drugs for various applications by implementing the CBQD platforms.

## 2. Materials and Methods

Paclitaxel > 99.5% purity and Tetrandrine > 98.0% purity were purchased from Fisher Scientific (Cayey, PR, USA) and used as is. Violacein was extracted from an environmental strain of *Chromobacterium violaceum* cultured on tryptic soy agar (TSA) at an ambient incubation temperature of 25 °C. The bacterial culture was centrifuged to obtain a pellet, which was then resuspended in methanol. The mixture was sonicated for 10 min, followed by a second round of centrifugation. The supernatant was transferred to a round-bottom flask for concentration. This procedure was repeated until the pellet was fully bleached, yielding 1.3 g of crude extract. A Soxhlet extraction was then performed using 250 mL of CHCl_3_ for 4 h. This was followed by an additional Soxhlet extraction with diethyl ether for 2 h. The process was repeated three times, with the final extraction carried out using 250 mL of ethanol to obtain an extract enriched in violacein. The final purification of violacein was accomplished using fast protein liquid chromatography (FPLC) with a Sephadex LH-20 column from GE HealthCare (Chicago, IL, USA). Forty grams of Sephadex LH-20 were packed into an XK16 column, and the compound was eluted with 100% methanol at a flow rate of 1 mL/min. The detection wavelength was set at 570 nm to accurately monitor violacein, and the purified fractions were collected using a FRAC-950 fraction collector from GE HealthCare (Chicago, IL, USA).

Nuclear magnetic resonance (NMR) characterizations were performed on a Bruker Ascend Aeon 500 (Billerica, MA, USA) using deuterium oxide (D_2_O) as a solvent. The solvent signals at 4.80 and 4.81 ppm were used as internal standards for protons. To study the morphology and dimensions of the nanocomposites, X-ray microanalyses were recorded with an FEI TALOS 200× high-resolution scanning transmission electron microscope (TEM) from FEI Company (Hillsboro, OR, USA). Transmission electron microscopy (TEM) and selected area electron diffraction (SAED) were performed using a FEI TALOS 200X high-resolution scanning/transmission electron microscope (TEM) from FEI Company (Hillsboro, OR, USA) at Brookhaven National Laboratory’s Center for Functional Nanomaterials under proposal number 312416. TEM and SAED images were processed using FIJI software (Version 1.54p) [27]. Powder diffractograms (P-XRD) were collected at 300 K using a Rigaku XtaLAB SuperNova X-ray diffractometer (The Woodlands, TX, USA) with a micro-focus Cu-Kα radiation (λ = 1.5417 Å) source and equipped with a HyPix3000 X-ray detector (50 kV, 1 mA). Powder samples were mounted on MiTeGen micro-loops and the powder diffractograms were collected between 0–60° using the Fast phi move experiment. Bragg’s law was used to calculate the d-spacings in the P-XRD spectra.

### 2.1. Synthesis of CBQDs and D-CBQDs

CBQDs and D-CBQDs where synthesized as reported in our previous work [8]. In a typical synthesis, 2 g of citric acid was placed into a beaker and heated to 200 °C using a heating mantle. The citric acid changes from colorless to yellow, and then orange. The orange color indicates the formation of CBQDs, which amounted to around 15 min of heating. The product was then solubilized in water and then filtered using a syringe filter with a pore size of 0.1 µm to remove any turbostratic graphene that may have formed as a side product. The synthesis for doped-CBQDs was done heating at 200 °C for 10 min 2 g of citric acid and 3-mercaptopropionic acid in a beaker to form sulfur-carbon based quantum dots (S-CBQDs). To prepare nitrogen-doped carbon-based quantum dots (N-CBQDs) 0.8 g of citric acid was mixed with NH_4_Cl in a beaker. The solution was heated at 200 °C for 10 min using a hotplate, and then the mixture changed from colorless to yellow and then orange, which indicated the formation of the product. The purification of the D-CBQDs is done through a 12 kDa dialysis bag for 1 h to remove residual reagents. After dialysis, the D-CBQDs were filtered using a syringe filter with a pore size of 0.1 µm to remove any turbostratic graphene that may have formed as a side product. This filtrate was then freeze-dried to yield a cookie-gel textured product.

### 2.2. Drug Loading on CBQDs and D-CBQDs

Drug loadings were prepared through a one-step process. CBQD and D-CBQD aqueous solutions were mixed with an ethanolic solution of each drug in a volume ratio of 1:1. The mixtures were incubated in an ultrasonic bath at 50 °C and roto evaporated to obtain the products. The respective drug on the obtained products was completely coupled and was then able to be dissolved in water.

### 2.3. Partition Coefficient Tests by NMR

The partition coefficient measurements were done in triplicate using NMR spectroscopy following the method proposed by Cumming and Rücker [28]. The method consisted of dissolving an amount of the sample in D_2_O in an NMR tube and obtaining an ^1^H-NMR spectrum. Next, an equal volume of 1-octanol was added to the NMR tube and the phases were mixed. The tube was left unperturbed for 1 h to allow the phases to separate and reach equilibrium. After the equilibrium time had passed, an NMR spectrum of the aqueous/lower phase was taken. Finally, a characteristic peak of the compound was integrated and normalized to the D_2_O peak in both spectra. The first spectrum provided ${RI}_{w}^{init}$, the second provided ${RI}_{w}^{equil}$, and *K_OW_* is calculated according to Equation (1):

(1) $$K_{ow}=\frac{{RI}_{w}^{init}-{RI}_{w}^{equil}}{{RI}_{w}^{equil}}$$

where ${RI}_{w}^{init}$ = relative NMR signal integration of the materials in D_2_O before equilibration with 1-octanol and ${RI}_{w}^{equil}$ = relative NMR signal integration of the materials in the aqueous phase after equilibration. The obtained *K*_ow_ is then utilized as the partition constant and its logarithmic value (Log *K*_ow_) is considered as the obtained Log P.

## 3. Results

### 3.1. Partition Coefficient (Log P)

**Figure 2:**
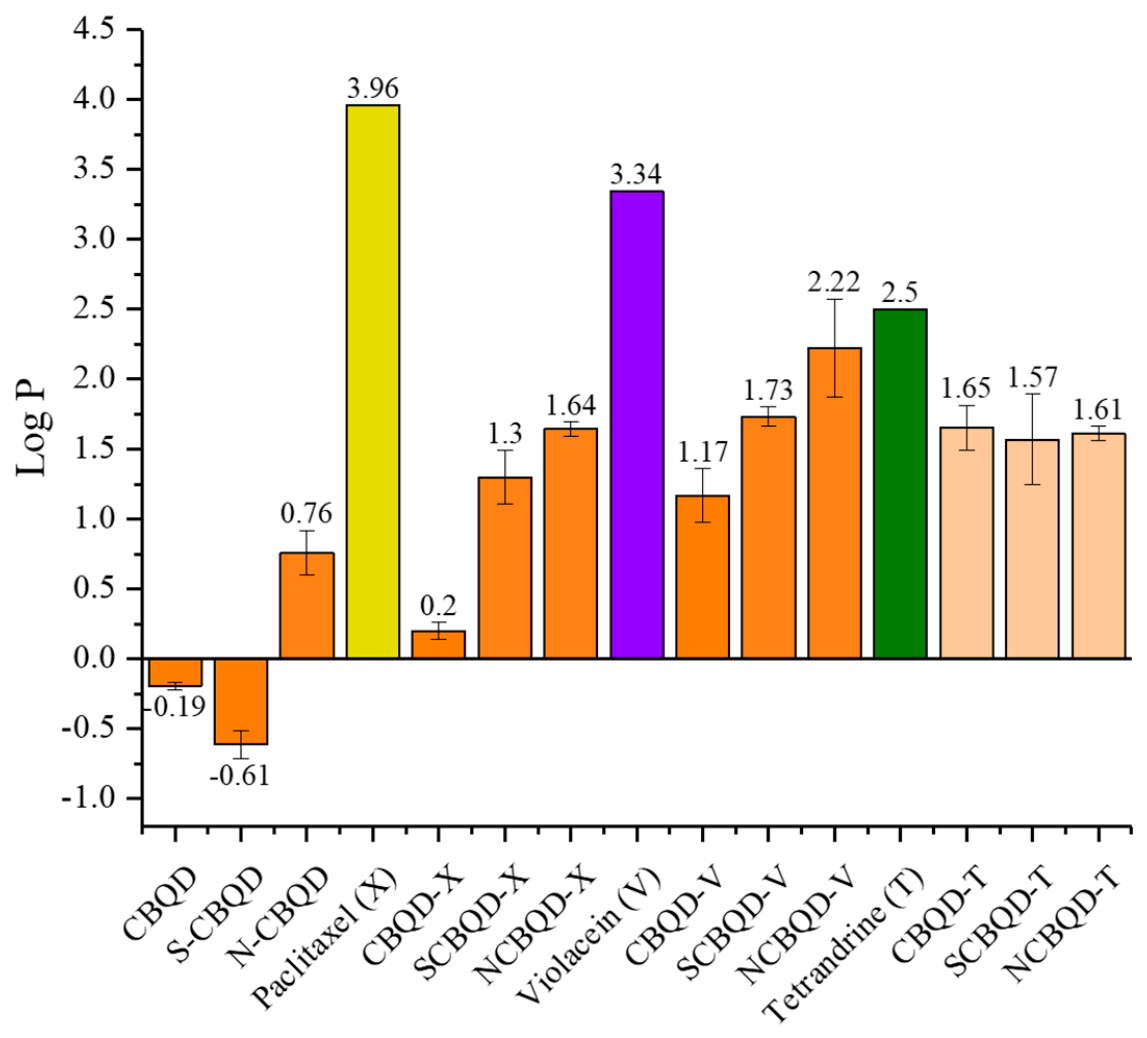
Partition coefficient results. The value of the Log P from the lone drugs was obtained from the published literature (Paclitaxel [25], Violacein [23] and Tetrandrine [13]).

Partition coefficient results (Table 1) showed a decrease in hydrophobicity in the three drugs. Tetrandrine showed a decrease of 0.85 logarithmic units in its Log P and minimal variation was observed between the doped and un-doped nanoparticles. Violacein was observed to lower its Log P (3.34) the most with CBQDs to 1.17, with S-CBQDs to 1.73 and to 2.22 with N-CBQDs (Figure 2). Violacein showed an improvement of around 1.12–2.17 logarithmic units, with CBQDs being the Log P that is lowered the most. Paclitaxel showed a dramatic shift in its Log P (3.96) to 0.20 with CBQDs, 1.30 with S-CBQDs and 1.64 with N-CBQDs. Paclitaxel became amphiphilic when loaded on CBQDs. Variance between signal peaks on the same spectrum could vary between 0.41–5.90%.

### 3.2. NMR Spectroscopy Coupling Analysis

#### 3.2.1. Paclitaxel

**Figure 3:**
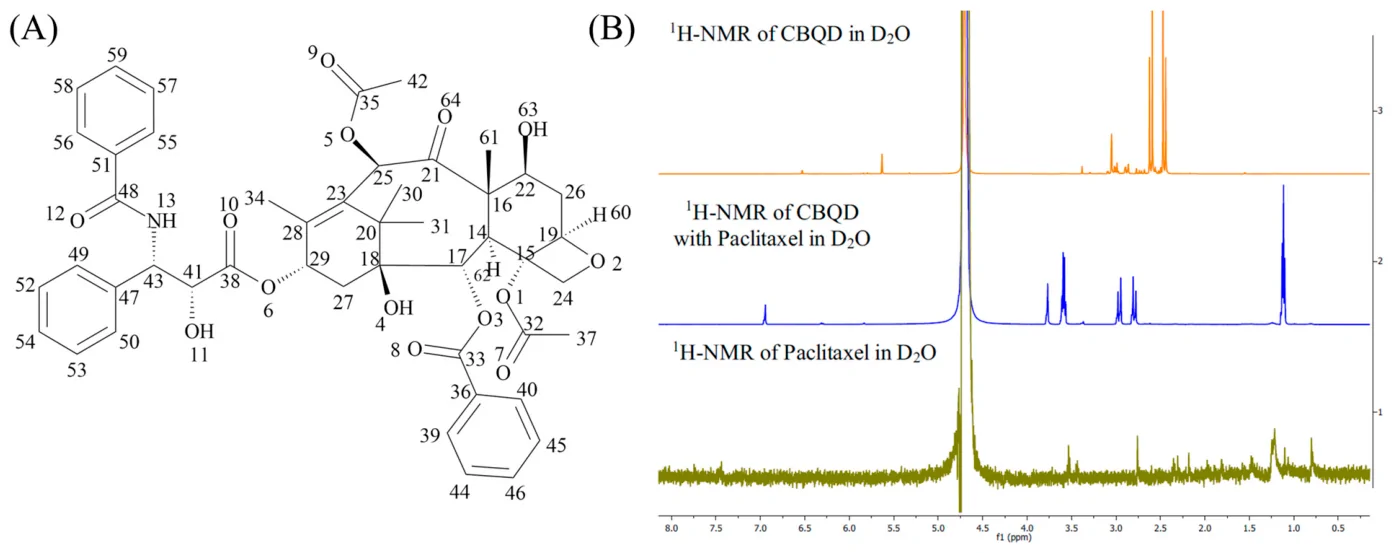
(**A**) Molecular structure of Paclitaxel with functional groups whose signals appeared after improvement in hydrophilicity. (**B**) ^1^H-NMR spectra comparing Paclitaxel, CBQD and CBQD with Paclitaxel (solvent D_2_O).

After ^1^H-NMR analysis, it was observed that the CBQD spectrum reflects a carbon nanoparticle with a graphitic core and functionalized edges. The 6.59 ppm, 5.95 ppm, and 5.87 ppm singlets belong to the sp^2^-hybridized aromatic and alkene protons directly on the graphitic core or at structural defect sites. The 2.58 ppm to 3.62 ppm singlets and doublets are sp^3^-hybridized aliphatic protons associated with oxygenated edge groups. Specifically, they represent protons adjacent to hydroxyls, epoxides, or ether linkages. The presence of doublets with the roof effect (3.34 ppm and 2.97 ppm) indicates rigidly held, strongly coupled protons on the functionalized edges. The peaks that appeared in Paclitaxel’s spectrum in D_2_O correspond to the aliphatic and acetate regions of the drug. The 3.53 ppm doublet corresponds to the oxygenated methine proton, typically from the carbon in the 14 position or 41 position (Figure 3) on the side chain. The 2.76 ppm singlet corresponds to a hydroxyl proton. The 2.31 and 2.18 ppm singlets correspond to the acetate methyl groups at the 15 and 25 positions of the taxane core. The 1.10 and 0.80 ppm singlets correlate to the tertiary methyl groups attached to the rigid baccatin III ring structure (like the 30, 31, or 61 positions’ methyls). The 1.22 ppm doublet is an aliphatic proton from the core or side chain experiencing localized coupling. When Paclitaxel is non-covalently coupled to the CBQDs, the NMR spectrum changes drastically. Uncoupled Paclitaxel features multiple aromatic signals from its phenyl rings well above 7.3 ppm [29]. However, in the coupled sample, the primary aromatic signals appear at 6.94 ppm, 6.31 ppm, and 5.83 ppm, distinct from the lone CBQD signals at 6.59 ppm, 5.95 ppm and 5.87 ppm. This dramatic upfield shift is the hallmark of π-π stacking. The triplet signal at 1.12 ppm corresponds to the methyl group of the traces of ethanol present in the sample. The paclitaxel aromatic rings are docking onto the flat surface of the CBQDs, sitting directly in the shielding cone generated by the CBQDs’ π delocalized-electron cloud.

Second-order coupling and roof effects were present across multiple peaks (6.94 ppm, 6.31 ppm, 5.83 ppm, and 1.27 ppm). The roof effect occurs when the difference in chemical shift (Δν) between two coupled protons becomes similar in magnitude to their coupling constant (J), causing the inner peaks of the multiplet to lean toward their coupling partner. In a non-covalent complex, the drug molecule loses its free rotational mobility. As Paclitaxel docks onto the CBQDs, it locks into a rigid conformation. Protons that were once dynamically averaged now act as a strongly coupled spin system because their relative spatial geometry is fixed.

The sharp acetate and tertiary methyl singlets from free Paclitaxel (the 2.31 ppm, 2.18 ppm, 1.10 ppm, and 0.80 ppm peaks) disappeared after the coupling with CBQDs. This is highly typical for nanoparticle–drug complexes. When a small molecule binds to a large nanoparticle, its tumbling rate in solution slows down drastically. This restricted movement leads to faster transverse relaxation (T2), which greatly broadens the peaks, often widening them enough that they merge into the baseline and disappear.

The peaks in S-CBQD reflect the functional groups on the edges and basal plane of the carbon dots, as well as any aliphatic remnants from the synthesis precursors. The 2.05 to 2.74 ppm singlets are characteristic of protons on carbons adjacent to heteroatoms (like the sulfur dopants or oxygen groups) or electron-withdrawing carbonyls and they also represent benzylic protons. The 1.82 ppm doublet of doublets and the 0.87 ppm triplet are signatures of a short aliphatic alkyl chain. The 0.87 ppm triplet is a terminal methyl group, and the 1.82 ppm signal corresponds to the adjacent methylene protons. When Paclitaxel was loaded onto S-CBQDs, the NMR spectrum did not just look like a simple addition of the two original spectra. New signals like the 6.93 ppm singlet corresponds to aromatic protons. The 3.75 ppm singlet and 3.60 ppm doublet of doublets correlate to the shifted oxygen-adjacent or nitrogen-adjacent protons from the paclitaxel side chains. Free paclitaxel has three bulky phenyl rings whose aromatic protons normally resonate downfield between 7.3 and 8.2 ppm. In the complex, these peaks are missing, but a new aromatic singlet appears at 6.93 ppm. This massive upfield shift (shielding) occurs because the paclitaxel phenyl rings are lying flat against the S-CBQD surface. The massive electron cloud of the graphene lattice creates a strong shielding cone, forcing the drug’s aromatic protons to resonate at a much lower frequency.

The differences between the isolated spectra and the complex confirm successful non-covalent loading. In the complex, several key peaks shift significantly to higher ppm values such as the N-CBQD aromatic peak at 6.70 ppm that shifts to 6.90 ppm. The isolated N-CBQD spectrum has signals at 6.52 ppm, 5.46 ppm, 5.31 ppm and 1.85 ppm that disappeared after coupling with paclitaxel. The isolated paclitaxel spectrum has an aliphatic signal at 2.18 ppm that disappeared in the complex’s spectrum. The N-CBQD spectrum features a distinct roof effect at 5.46/5.31 ppm (doublet of doublets). In the complex, this roof effect is preserved at 6.29/5.81 ppm (as doublets). The preservation of this coupling pattern proves that the structural integrity of the N-CBQD edges remains intact, but the loss of the finer splitting (dd becoming d) further supports the restriction of mobility and slight line broadening caused by the bulky Paclitaxel molecules docking onto the N-CBQDs’ surface.

#### 3.2.2. Violacein

**Figure 4:**
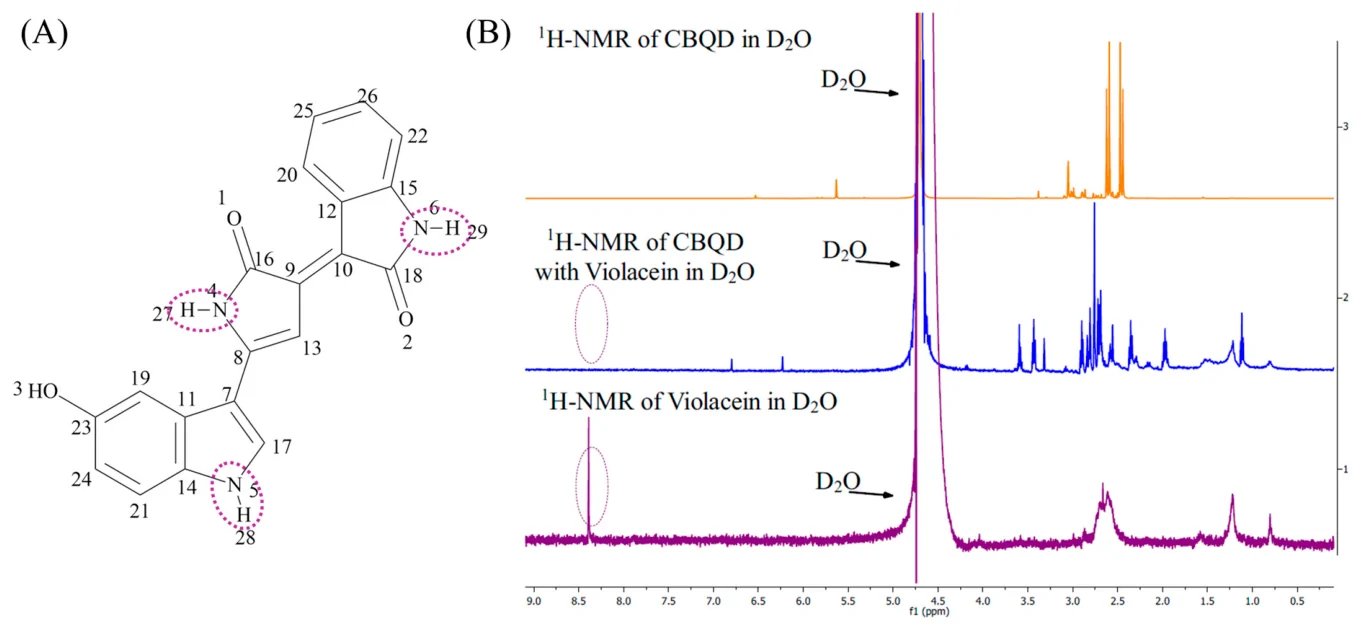
(**A**) Molecular structure of Violacein with functional groups whose signals disappeared due to Nuclear Overhauser Effect (NOE). Purple circles highlight the hydrogen placements whose signal disappeared after loading onto CBQDs. (**B**) ^1^H-NMR spectra comparing Violacein, CBQD and CBQD with Violacein (solvent D_2_O). Purple circles highlight a signal peak corresponding to Violacein that disappeared after loading onto CBQDs.

The ^1^H-NMR spectra of violacein loaded on the CBQDs revealed the disappearance of two distinctive peaks of violacein. The first was at 8.39 ppm, which corresponds to the hydrogen directly bound to the nitrogen in the lactam functional group in the structure of the drug, and the second was peaks that range from 2.76 to 2.53 ppm corresponding to a proton from the Ph-CH_2_-CH_3_ functional group (Figure 4). This trend was observed in all three nanoparticles loaded with Violacein. This finding suggests a strong affinity and binding between the lactam proton and the aromatic components of the nanoparticle.

We observed that the vinylic protons of the CBQD that range from 6.53 to 5.32 ppm disappear in the loaded spectra (Figure 4). The Violacein-loaded nanoparticle spectra showed two peaks unique to the drug-loaded nanoparticle at 6.8 ppm and 6.23 ppm (Table S4). These peaks can be attributed to aromatic loadings within the nanoparticle system. The presence of these peaks further supports the presence of interactions between the aromatic groups of Violacein and the CBQDs/D-CBQDs.

The lactam peak at 8.39 ppm of Violacein was not present in the spectrum of the drug-loaded S-CBQD [22]. This reveals these groups interact with S-CBQD. The vinylic protons’ peak present in the S-CBQD range from 5.87 ppm to 5.26 ppm disappears in the spectra of the drug-loaded nanoparticle, signifying interaction with the drug (Table S5). Other peaks that disappear include a singlet peak of a proton in the alpha position to a sulfur atom in a high-tension ring at 2.74 ppm, protons on a methyl of thioacetone at 2.51, and protons from methyl connected to double bonds at 2.51 ppm–1.55 ppm.

The N-CBQD peaks at 8.39 ppm, 6.93 ppm, related to an ortho H of a phenol with an electron donation group, 6.33–5.84 ppm vinylic protons, 3.76 ppm corresponding to an alpha proton of alcohol, and 3.39–1.94 ppm from protons of a double bounded to an electron-withdrawing group disappeared when loaded with Violacein, indicating participation in the interactions with the coupled drug (Table S6).

#### 3.2.3. Tetrandrine

How Tetrandrine couples on CBQDs is crucial to understanding the level of improvement in hydrophilicity. Through ^1^H-NMR analysis, the positions of Tetrandrine bound on the CBQDs can be elucidated.

**Figure 5:**
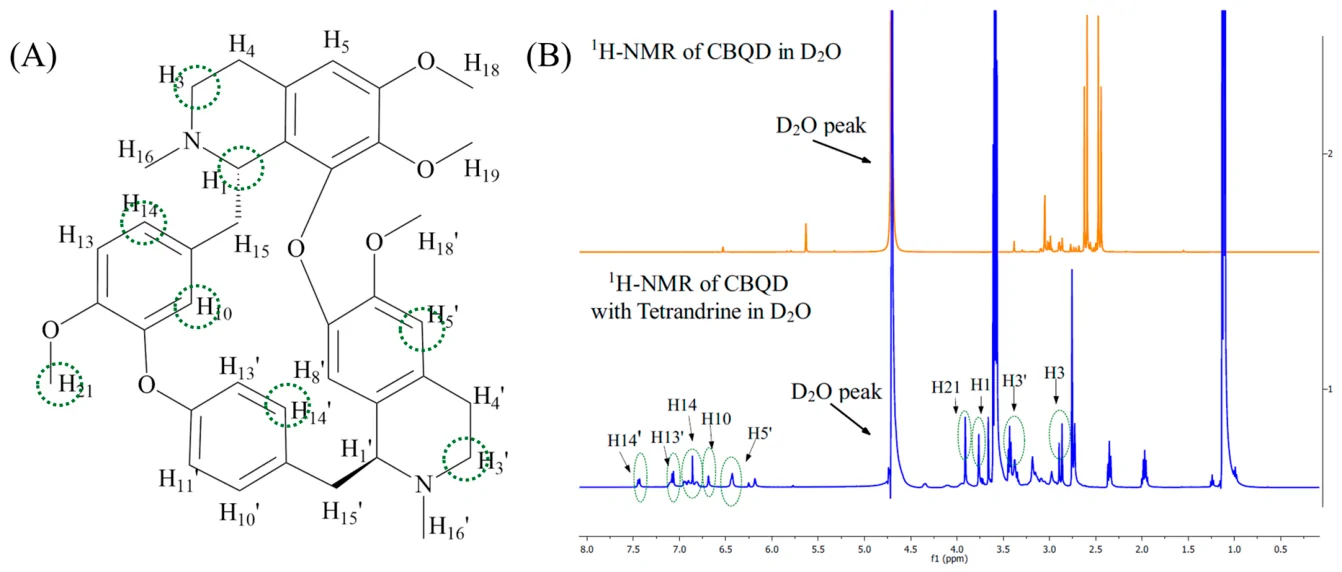
(**A**) Molecular structure of Tetrandrine with functional groups whose signals did not disappear due to Nuclear Overhauser Effect (NOE) although exhibiting a frequency shift. Green circles highlight the hydrogen placements whose signal did not disappear after loading onto the CBQDs. (**B**) ^1^H-NMR spectra comparing CBQD and CBQD with Tetrandrine (solvent D_2_O). Green circles highlight the signals corresponding to Tetrandrine that did not disappear after loading onto the CBQDs.

Examining the binding relationship between the CBQDs and Tetrandrine, we can determine that the binding of the molecule was due to the absence and presence of specific aromatic regions. The absence of H5 indicates a coupling of an aromatic region of Tetrandrine to the CBQD. Protons H11′, H10′, and H1′ are additionally not present after the binding to the CBQD. Since H1′ is a stereocenter, this indicates the side of the drug that faces against the CBQD’s surface when coupling. The absence of specific aromatic peaks in 5–7 ppm corresponded to the binding of CBQDs and Tetrandrine. ^1^H-NMR demonstrated the presence of new peaks at 6.84 ppm (Figure 5).

The presence and shift of protons H18′, H13′, and H14′ further supported the binding of Tetrandrine to the CBQDs. The new peaks at 5.77 ppm correspond to the coupling of tetrandrine to the CBQDs (Table S1). The alkyl protons indicate the parallel binding of tetrandrine CBQDs and are specific to the loading of Tetrandrine and the CBQDs. The presence of H13′ and H14′ highlights the conclusion that an aromatic region is bound to the CBQDs and their derivatives. These peaks are shifted from the original ^1^H-NMR spectrum of Tetrandrine, and the shift corresponds to the binding relationship [30]. The absence of multiple Tetrandrine aromatic overlapping demonstrates that the binding is not a mixture of various binding positions when compared to the ^1^H-NMR spectra of solely Tetrandrine [31]. The CBQDs and their doped derivatives all indicated this trend and show similar binding (Figure 5). The absence of the peaks correlates to the coupling of the drug to the CBQD. The ^1^H-NMR indicates that protons H18 and H21 are present, while there is an absence of protons H19 and H16′ which are concurrent with the molecular structure of Tetrandrine.

The ^1^H-NMR loading of Tetrandrine and S-CBQD demonstrated the absence of S-CBQD peaks, which correlates to the coupling. With the knowledge that the Tetrandrine had bound to the S-CBQD, the presence and absence of methyl and aromatic regions in the ^1^H-NMR allowed for the conclusion that there was binding between the CBQDs and Tetrandrine (Table S2). Table S3 illustrates the presence of specific functional groups, providing an accurate depiction of how Tetrandrine is bonded in all three derivatives. The bonding pattern of Tetrandrine loaded onto N-CBQD followed a trend similar to that observed in CBQD and S-CBQDs (Table 2).

### 3.3. Selected Area Electron Diffraction (SAED) and Powder X-Ray Diffraction (P-XRD)

**Figure 6:**
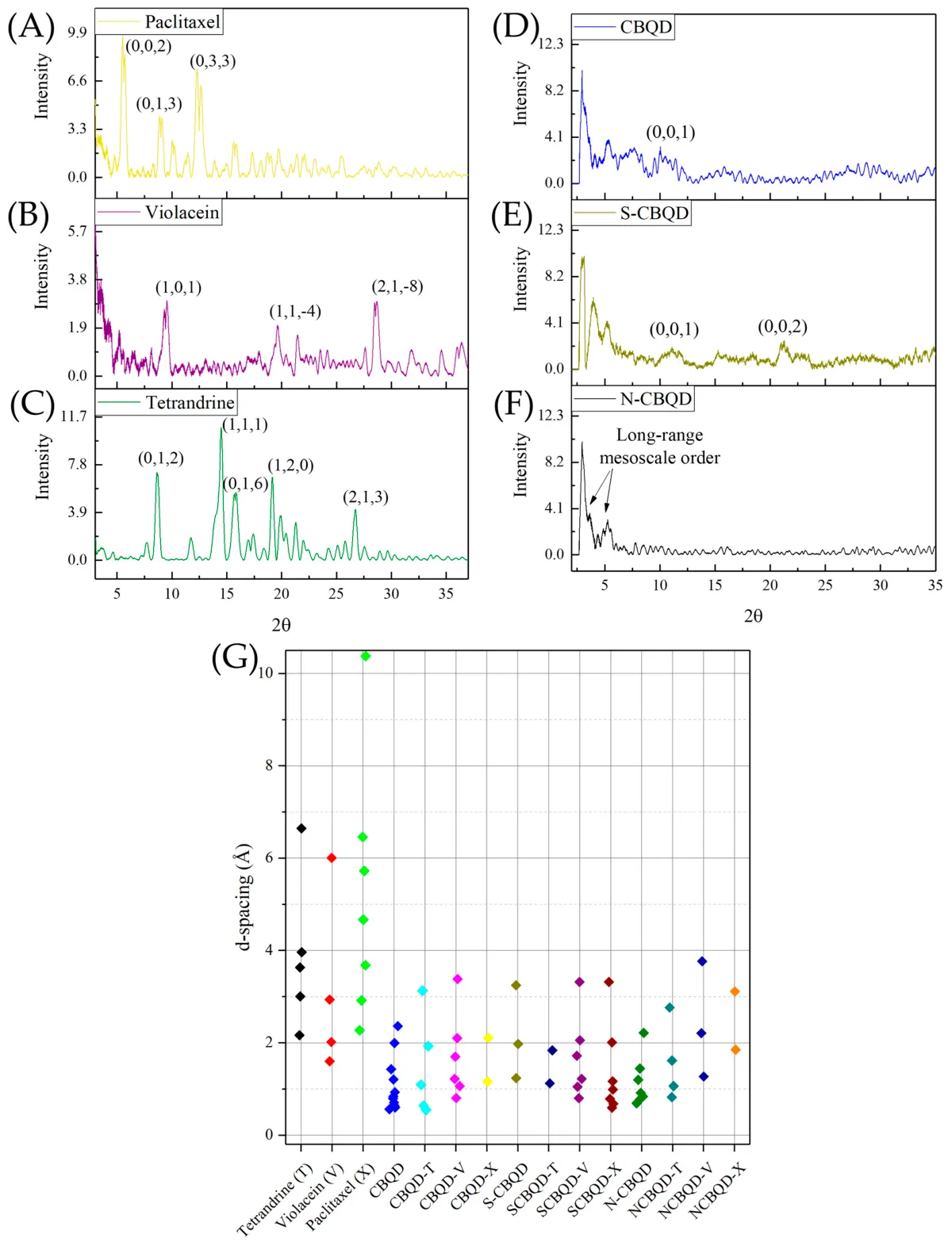
(**A**) P-XRD spectrum of Paclitaxel, (**B**) P-XRD spectrum of Violacein, (**C**) P-XRD spectrum of Tetrandrine, (**D**) P-XRD spectrum of CBQD, (**E**) P-XRD spectrum of S-CBQD, (**F**) P-XRD spectrum of N-CBQD and (**G**) column scatter graph comparing d-spacings in Å between lone drugs, lone nanomaterials and drug-loaded nanomaterials (values in Table S11). Spectra may be compared to the literature P-XRDs for Paclitaxel [34], Violacein [35,36,37] and Tetrandrine [32].

**Figure 7:**
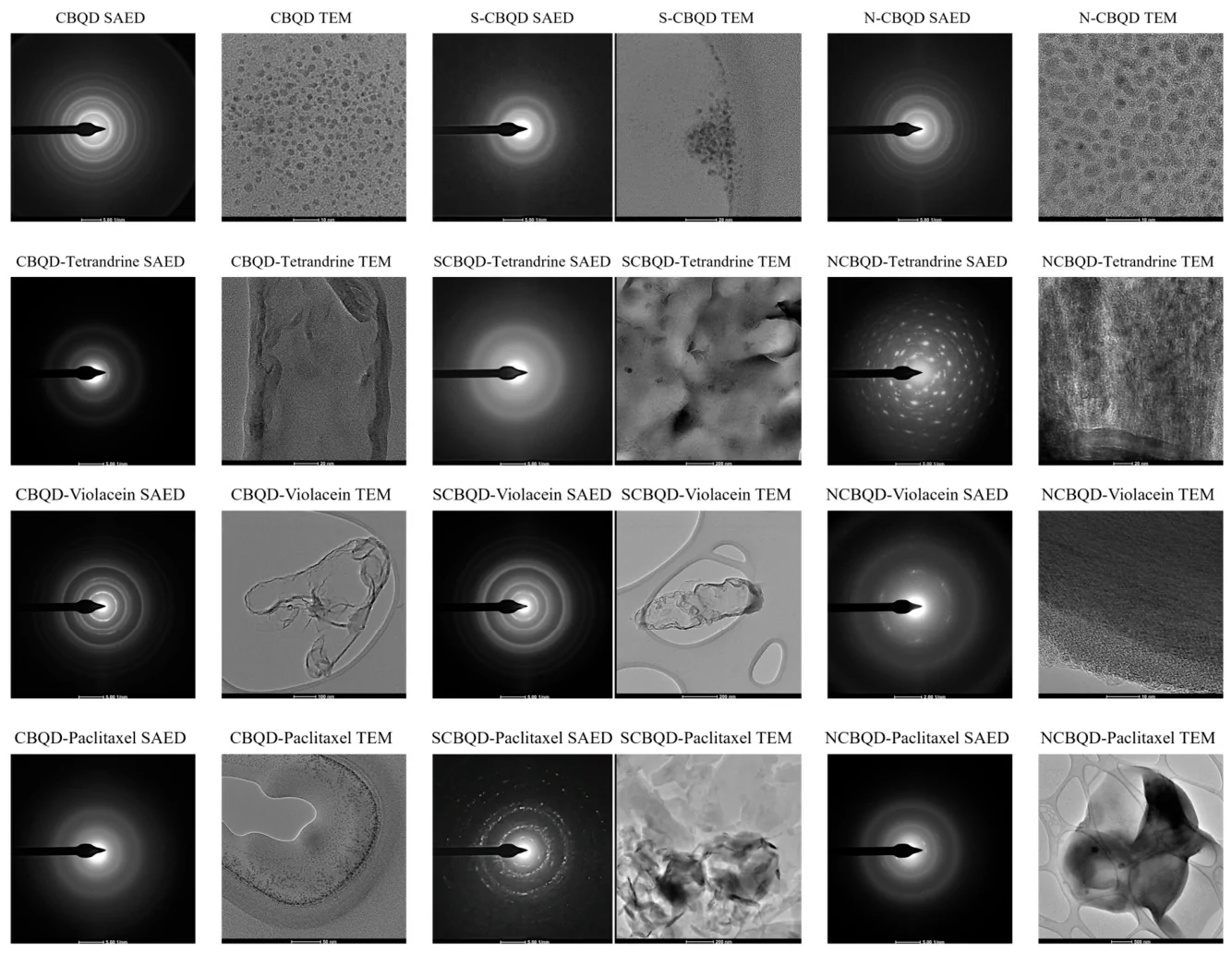
SAED patterns of nanomaterials and drug-loaded nanomaterials and their respective TEM images.

After loading the drugs on the nanomaterials, the product had a gel-like consistency which made analysis via P-XRD unsuitable at room temperature. For this reason, SAED was performed to observe possible changes in the d-spacings of the nanoparticles and the nanoparticles loaded with the drugs. Results showed differences in the crystal lattice between the lone reagents and the drug-loaded nanomaterials (see Figure 6). This confirms that there are changes in the crystal lattice when coupling the nanomaterials and the drugs. With both CBQDs and S-CBQDs, their P-XRD spectra showed bands of oxidized graphitic structures and SAED confirmed the presence of a long-range order (see Figure 7 for SAED patterns and TEM). In N-CBQD, SAED confirmed that the values shown in its P-XRD spectrum uncover a long-range order. Observing no other bands in N-CBQDs’ P-XRD spectrum confirms that the sample is a successfully isolated carbon-based quantum dot product without unwanted crystalline byproducts or restacked graphitic agglomerates [33].

## 4. Discussion

Improvement in hydrophilicity was observed in the three studied drugs when coupled to the CBQDs and D-CBQDs. The water solubilization effect was observed on all the drugs added to the mixture of CBQDs/D-CBQDs, and the coupling product had a gel texture after being lyophilized. The Log P improvement effect was limited on the solvent to solute ratio because of the initial solubility of the drug before coupling with the nanoparticles. Further NMR data analysis concluded that the Tetrandrine had coupled concave down against the nanoparticles’ surface. This may be due to the angle bond strain in Tetrandrine’s molecular geometry. Observations lead to the conclusion that Violacein was loaded onto the three nanoparticles’ surface in a comparable manner, i.e., lying flat. The aromatic groups in Paclitaxel appear to have a crucial role in the coupling to the nanoparticles’ surface. This seems to have enough interaction to provide considerable improvement in hydrophilicity even with many Paclitaxel signals still present in the spectra of the drug loaded on the nanoparticles. Observed changes in d-spacing values confirmed the drug–nanoparticle couplings also affect the crystal lattice. The CBQDs act as dynamic, amphiphilic molecular bridges rather than passive carriers, playing an active, three-fold role in the solubilization mechanism. First, the sp2-hybridized carbon domains within the carbon dots’ cores serve as hydrophobic anchors, engaging in strong π-π stacking and van der Waals interactions with the planar aromatic rings of the drug molecules. Second, the carbon dots disrupt drug aggregation by competitively binding to the drug molecules; the carbon dots outcompete the innate intermolecular drug–drug interactions, preventing precipitation. Finally, the heavily functionalized carbon dot surface, enriched with high-polarity defect sites driven by the nitrogen or sulfur atom dopants, creates a dense network of electrostatic and hydrogen-bonding interactions with water. Through this synergistic mechanism, the carbon dots couple with the hydrophobic drug moieties while presenting a highly hydrophilic exterior shield, thermodynamically driving the Log P reduction observed. While this study establishes the fundamental physical chemistry and theoretical structural mechanisms driving solubilization, specifically the interplay of π-π stacking and dopant-induced hydrogen bonding, fully mapping the thermodynamics of these interactions remains an ongoing objective. Future studies will incorporate molecular dynamics simulations to visualize the atomistic host–guest interactions, calculate precise binding free energies, and model the explicit hydration shells at the CBQD–drug interface, thereby computationally validating the proposed amphiphilic bridging mechanism.

Overall, the efficiency of lowering a drug’s Log P was affected by steric factors, including the drug’s chirality, and the nanoparticle’s surface polarity. These results show that anti-cancer drugs’ hydrophobicity can be tuned without the use of toxic organic solvents, without drug encapsulation or structural modification and using just small amounts of nanoparticle to lower the partition coefficient. This improvement in the drugs’ hydrophilicity may enhance the effectiveness of these drugs as intravenous therapies, an application that typically requires drugs to be water soluble [38]. In addition, the improvement in hydrophilicity’s applications may be expanded to fields such as water and soil remediation of hydrophobic contaminants [39], or to enhance the dispersion of incompatible components in polymers or coatings [40], among others.

## 5. Conclusions

The hydrophobicity of Tetrandrine, Violacein and Paclitaxel was able to be lowered using CBQDs, S-CBQDs and N-CBQDs; the reduction reflects the apparent or effective partitioning behavior of the formulated CBQD/D-CBQD–drug complex. The way the drugs positioned themselves on the nanoparticles’ surface was analyzed via ^1^H-NMR and factors that affected the efficiency of lowering the Log P were identified. In addition to the role of the drugs’ capacity to be in contact with the nanomaterials, there was an observed effect in the efficiency of lowering the Log P as the electronegativity and atomic radii of the doping atom were changed. These differences were more noticeable when coupling the nanomaterials with Paclitaxel and Violacein than Tetrandrine. This feature appeared in the drugs that had more access to the nanomaterials’ surface due to the drugs’ molecular geometry, hinting that this feature appears as a secondary effect once an interlocking capacity has been achieved. These results show the possibility of improving the hydrophilicity of a wider variety of drugs and more effective treatments at lower dosages and thus reduced side effects. It is known that the ability of nanosystems to reach their target without causing toxicity can be enabled by the hydrophilicity and hydrophobicity of their surfaces, along with other external factors [41]. Previous studies have shown that systems similar to CBQDs can lower the IC_50_ of drugs against cancer cells and in mouse models [42,43,44,45,46]. However, there is a lack of literature where the Log P is measured after the loading or encapsulation process is done. Limitations of the NMR method include less sensitivity in comparison to methods like high-performance liquid chromatography and mass spectrometry, with the NMR method needing concentrations in micromolar scale for effective detection. Other limitations are shared with the shake flask method such as temperature and pH sensitivity, vulnerability to emulsions and range of measurable Log P (often between −2 to 4) [47]. Therefore, future work will involve confirming the effect of lowering the Log P of hydrophobic drugs via pharmacokinetic studies (absorption, distribution, metabolism, and excretion) [48] via in vitro and in vivo models comparing the original drugs’ activity with and without the CBQDs.

## 6. Patents

Patent (PCT) publication number WO2024206177.

## Figures and Tables

**Table 1:** Partition coefficient results.

Sample Name	Log P 1	Log P 2	Log P 3	Average	Std. Dev.
CBQD [8]	−0.1769	−0.2230	−0.1782	−0.1927	0.0262
S-CBQD [8]	−0.4940	−0.6879	−0.6593	−0.6138	0.1047
N-CBQD [8]	0.9361	0.7118	0.6303	0.7594	0.1584
Tetrandrine (T)	2.5 [13]				
CBQD-T	1.5093	1.8191	1.6295	1.6526	0.1562
SCBQD-T	1.1984	1.7946	1.7093	1.5674	0.3224
NCBQD-T	1.6656	1.6168	1.5603	1.6142	0.0527
Violacein (V)	3.34 [23]				
CBQD-V	1.3856	1.0224	1.1150	1.1743	0.1887
SCBQD-V	1.8156	1.7216	1.6704	1.7359	0.0737
NCBQD-V	1.8469	2.5425	2.2845	2.2246	0.3517
Paclitaxel (X)	3.96 [25]				
CBQD-X	0.2722	0.1514	0.1768	0.2001	0.0637
SCBQD-X	1.4611	1.3601	1.0926	1.3046	0.1904
NCBQD-X	1.6215	1.7098	1.6107	1.6473	0.0544

**Table 2:** Correlation of Log P improvements and drug–nanoparticle coupling characteristics.

Drug	Platform	Drugs’ Original Log P	Log P	Observations	3D Visualizer
Paclitaxel	CBQD	3.96 [25]	0.2	Paclitaxel has many flexible aromatic groups that can make π-π stacking interactions. It is observed that the lowest Log P is achieved when coupled with the CBQD, which does not have hetero atoms modifying the surface’s aromaticity.	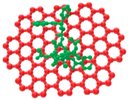
	S-CBQD		1.3		
	N-CBQD		1.64		
Violacein	CBQD	3.34 [23]	1.17	Violacein has polar functional groups that may cause repulsion with the surface of the nanoparticles. It is observed that the nanoparticle that provided the least improvement was the N-CBQD which has the more electronegative, therefore more polar, hetero atom doping.	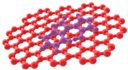
	S-CBQD		1.73		
	N-CBQD		2.22		
Tetrandrine	CBQD	2.5 [13]	1.65	In Tetrandrine’s case, even with aromatic groups, a certain lowering of its Log P was observed but no differences from hetero atom doping were observed. This indicates that the drug’s molecular geometry was key in its ability to couple to the nanoparticles’ surface. Tetrandrine has a slight concavity in its shape, as observed in its crystal structure [32].	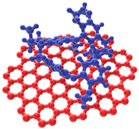
	S-CBQD		1.57		
	N-CBQD		1.61		

## Data Availability

The original contributions presented in this study are included in the article/Supplementary Materials. Further inquiries can be directed to the corresponding authors.

## Supplementary Materials

The following supporting information can be downloaded at https://www.mdpi.com/article/10.3390/nano16181166/s1, Table S1: ^1^H-NMR (500 MHz, D_2_O) summary of the binding of Tetrandrine with CBQDs; Table S2: ^1^H-NMR (500 MHz, D_2_O) summary of the binding of Tetrandrine with S-CBQDs; Table S3: ^1^H-NMR (500 MHz, D_2_O) summary of the binding of Tetrandrine with N-CBQDs; Table S4: ^1^H-NMR (500 MHz, D_2_O) summary of the binding of Violacein with CBQDs; Table S5: ^1^H-NMR (500 MHz, D_2_O) summary of the binding of Violacein with S-CBQDs; Table S6: ^1^H-NMR (500 MHz, D_2_O) summary of the binding of Violacein with N-CBQDs; Table S7: ^1^H-NMR (500 MHz, D_2_O) summary of the binding of Paclitaxel with CBQDs; Table S8: ^1^H-NMR (500 MHz, D_2_O) summary of the binding of Paclitaxel with S-CBQDs; Table S9: ^1^H-NMR (500 MHz, D_2_O) summary of the binding of Paclitaxel with N-CBQDs; Table S10: d-spacings of lone drugs, nanomaterials and drug-loaded nanomaterials; Table S11: d-spacings of lone drugs, nanomaterials and drug-loaded nanomaterials; Figure S1: ^1^H-NMR of Tetrandrine in D_2_O; Figure S2: ^1^H-NMR of Violacein in D_2_O; Figure S3: ^1^H-NMR of Paclitaxel in D_2_O; Figure S4: ^1^H-NMR of CBQD in D_2_O; Figure S5: ^1^H-NMR of S-CBQD in D_2_O; Figure S6: ^1^H-NMR of N-CBQD in D_2_O; Figure S7: ^1^H-NMR of CBQD-Tetrandrine in D_2_O; Figure S8: ^1^H-NMR of SCBQD-Tetrandrine in D_2_O; Figure S9: ^1^H-NMR of NCBQD-Tetrandrine in D_2_O; Figure S10: ^1^H-NMR of CBQD-Violacein in D_2_O; Figure S11: ^1^H-NMR of SCBQD-Violacein in D_2_O; Figure S12: ^1^H-NMR of NCBQD-Violacein in D_2_O; Figure S13: ^1^H-NMR of CBQD-Paclitaxel in D_2_O; Figure S14: ^1^H-NMR of SCBQD-Paclitaxel in D_2_O; Figure S15: ^1^H-NMR of NCBQD-Paclitaxel in D_2_O.

## Abbreviations

The following abbreviations are used in this manuscript:

Log P	Partition coefficient
CBQDs	Carbon-based quantum dots
D-CBQDs	Doped carbon-based quantum dots
S-CBQDs	Sulfur-doped carbon-based quantum dots
N-CBQDs	Nitrogen-doped carbon-based quantum dots
X	Paclitaxel
V	Violacein
T	Tetrandrine
^1^H-NMR	Proton nuclear magnetic resonance
NOE	Nuclear Overhauser Effect
TEM	Transmission electron microscopy
SAED	Selected area electron diffraction
P-XRD	Powder X-ray diffraction
TSA	Tryptic soy agar
FPLC	Fast protein liquid chromatography
