# Supplementary material for: Improving the Hydrophilicity of the Drugs Paclitaxel, Violacein, and Tetrandrine Using Carbon-Based Quantum Dots
Source: Nanomaterials (Basel). 2026 Sep 16;16(18):1166. doi: 10.3390/nano16181166 (PMC13610360; doi:10.3390/nano16181166)
Supplement: Supplementary file 1 [file nanomaterials-16-01166-s001.zip › nanomaterials-4516204-supplementary.pdf]

Supporting Information

Improving the Hydrophilicity of the Drugs Paclitaxel, Violacein, and Tetrandrine Using Carbon-Based Quantum Dots

Christine Wu [1,†], Katherine Ayala [2,†], Solimar Collazo-Hernández [3], MiaSara Pérez-Salvá [3], Nataniel Medina-Berríos [4,5,*], Fabiola M. Rosa-Suárez [3], Alondra Veloz-Bonilla [3], Rafael A. Villanueva-Nogueras [3], Alexis Lavín Flores [4], Keyla A. López-Pérez [4], Valerie Ortiz-Gómez [6], Luis Prieto-Costas [7], Rafael Maldonado-Hernández [8], Marvin J. Bayro [4], Gerardo Morell [9] and Brad R. Weiner [4,*]

[1] Department of Chemistry, Smith College, Northampton, MA 01063, USA; wu.christinemass@gmail.com
[2] Department of Chemistry, Berea College, Berea, KY 40404, USA; ayalak@berea.edu
[3] Department of Biology, University of Puerto Rico-Río Piedras Campus, San Juan, PR 00925, USA; solimar.collazo12@gmail.com (S.C.-H.); miasara.perez@upr.edu (M.P.-S.); fabiola.rosa3@upr.edu (F.M.R.-S.); alondra.veloz@upr.edu (A.V.-B.); rafael.villanueva1@upr.edu (R.A.V.-N.)
[4] Department of Chemistry, University of Puerto Rico-Río Piedras Campus, San Juan, PR 00925, USA; alexis.lavin@upr.edu (A.L.F.); keyla.lopez1@upr.edu (K.A.L.-P.); marvin.bayro@upr.edu (M.J.B.)
[5] National Coalition of Independent Scholars, Brattleboro, VT 05301, USA
[6] School of Dental Medicine, Surgical Sciences Department, University Ana G. Méndez Gurabo Campus, Gurabo, PR 00777, USA; ortizv3@uagm.edu
[7] Center for Tropical Biodiversity, Puerto Rico Science Trust, San Juan, PR 00936, USA; lprieto@prsciencetrust.org
[8] Department of Biology, University of Puerto Rico-Cayey Campus, Cayey, PR 00736, USA; rafael.maldonado1@upr.edu
[9] Department of Physics, University of Puerto Rico-Río Piedras Campus, San Juan, PR 00925, USA; gerardo.morell@upr.edu
* Correspondence: nataniel.medina@upr.edu (N.M.-B.); brad.weiner@upr.edu (B.R.W.)
† These authors contributed equally to this work.

Table of contents:

Table S1: ^{1}H-NMR (500 MHz, D_2O) summary of the binding of Tetrandrine with CBQDs.

Table S2: ^{1}H-NMR (500 MHz, D_2O) summary of the binding of Tetrandrine with S-CBQDs.

Table S3: ^{1}H-NMR (500 MHz, D_2O) summary of the binding of Tetrandrine with N-CBQDs.

Table S4: ^{1}H-NMR (500 MHz, D_2O) summary of the binding of Violacein with CBQDs.

Table S5: ^{1}H-NMR (500 MHz, D_2O) summary of the binding of Violacein with S-CBQDs.

Table S6: ^{1}H-NMR (500 MHz, D_2O) summary of the binding of Violacein with N-CBQDs.

Table S7: ^{1}H-NMR (500 MHz, D_2O) summary of the binding of Paclitaxel with CBQDs.

Table S8: ^{1}H-NMR (500 MHz, D_2O) summary of the binding of Paclitaxel with S-CBQDs.

Table S9: ^{1}H-NMR (500 MHz, D_2O) summary of the binding of Paclitaxel with N-CBQDs.

Table S10: Partition coefficient results.

Table S11: d-spacings of lone drugs, nanomaterials and drug-loaded nanomaterials.

Figure S1: ^{1}H-NMR of Tetrandrine in D_2O.

Figure S2: ^{1}H-NMR of Violacein in D_2O.

Figure S3: ^{1}H-NMR of Paclitaxel in D_2O.

Figure S4: ^{1}H-NMR of CBQD in D_2O.

Figure S5: ^{1}H-NMR of S-CBQD in D_2O.

Figure S6: ^{1}H-NMR of N-CBQD in D_2O.

Figure S7: ^{1}H-NMR of CBQD-Tetrandrine in D_2O.

Figure S8: ^{1}H-NMR of SCBQD-Tetrandrine in D_2O.

Figure S9: ^{1}H-NMR of NCBQD-Tetrandrine in D_2O.

Figure S10: ^{1}H-NMR of CBQD-Violacein in D_2O.

Figure S11: ^{1}H-NMR of SCBQD-Violacein in D_2O.

Figure S12: ^{1}H-NMR of NCBQD-Violacein in D_2O.

Figure S13: ^{1}H-NMR of CBQD-Paclitaxel in D_2O.

Figure S14: ^{1}H-NMR of SCBQD-Paclitaxel in D_2O.

Figure S15: ^{1}H-NMR of NCBQD-Paclitaxel in D_2O.

Tetrandrine ^{1}H-NMR Tables

Table S1: ^{1}H-NMR (500 MHz, D_2O) summary of the binding of Tetrandrine with CBQDs.

	Signals from CBQD (ppm)	Signals from Tetrandrine (ppm)	Signals from CBQD-Tetrandrine (ppm)
Disappeared	6.98 (s), 6.62 (s), 6.53, 6.47, 5.84 (s), 5.84 (s) 5.80 (s), 5.63 (s), 5.46, 5.33 (s), 3.29, 3.05-2.95 (m). 2.62-2.43 (m), 2.17 (s), 1.86 (s), 1.19 (s),	6.55 (s), 5.99 (s), 2.62 (s, 3H)	N/A
Appeared new	N/A	N/A	6.84 (s) 6.69 (s), 6.42 (s), 5.77 (s), 4.35 (d), 4.10 (s)

Table S2: ^{1}H-NMR (500 MHz, D_2O) summary of the binding of Tetrandrine with S-CBQDs.

	Signals from S-CBQD (ppm)	Signals from Tetrandrine (ppm)	Signals from SCBQD-Tetrandrine (ppm)
Disappeared	6.10 (s), 5.87 (s), 5.67 (s), 2.52-0.0	6.55 (s), 5.99 (s), 2.62 (s)	N/A
Appeared new	N/A	N/A	6.69 (s), 5.74 (s), 4.35(d), 4.10 (s), 3.63 (s), 2.98 (s), 2.35 (t), 0.97 (tt)

Table S3: ^{1}H-NMR (500 MHz, D_2O) summary of the binding of Tetrandrine with N-CBQDs.

	Signals from N-CBQD (ppm)	Signals from Tetrandrine (ppm)	Signals from NCBQD-Tetrandrine (ppm)
Disappeared	6.94 (s)	5.99 (s), 6.29 (s), 6.30 (s), 6.55 (s), 3.88 (s), 2.33 (s)	N/A
Appeared new	N/A	N/A	6.69 (s) 6.44 (s), 5.70(s), 4.36 (s), 4.10 (s), 2.98 (s), 3.17 (s), 1.12 (t)

Violacein ^{1}H-NMR Tables

Table S4: ^{1}H-NMR (500 MHz, D_2O) summary of the binding of Violacein with CBQDs.

	Signals from CBQD (ppm)	Signals from Viola-cein (ppm)	Signals from CBQD-Violacein (ppm)
Disappeared	6.53 (s), 5.84 (s), 5.8 (s), 5.63 (s), 5.32 (s)	8.39 (s)	N/A
Appeared new	N/A	N/A	6.8 (s), 6.23 (s), 3.61-3.56 (m), 3.45-3.41 (t), 3.32 (s), 2.90-2.55 (m)

Table S5: ^{1}H-NMR (500 MHz, D_2O) summary of the binding of Violacein with S-CBQDs.

	Signals from S-CBQD (ppm)	Signals from Viola-cein (ppm)	Signals from SCBQD-Violacein (ppm)
Disappeared	5.87 (s), 5.26 (s), 2.74 (s), 2.51 (m), 2.14 (bs), 1.95 (t), 1.81 (dd), 1.55 (t).	8.39 (s), 2.76-2.53 (d)	N/A
Appeared new	N/A	N/A	6.75 (s), 3.55 (s), 3.44 (t), 2.90 (t), 2.77 (m), 2.66 (m), 2.36 (t), 1.98 (m), 1.12 (t)

Table S6: ^{1}H-NMR (500 MHz, D_2O) summary of the binding of Violacein with N-CBQDs.

	Signals from N-CBQD (ppm)	Signals from Violacein (ppm)	Signals from NCBQD-Violacein
Disappeared	6.93 (s), 6.33 (s), 5.84(d), 3.76 (s), 3.39-1.94.	8.39 (s), 2.76-2.53 (d)	N/A
Appeared new	N/A	N/A	3.61-3.56 (q), 3.45-3.41 (t), 1.12 (t)

Paclitaxel ^{1}H-NMR Tables

Table S7: ^{1}H-NMR (500 MHz, D_2O) summary of the binding of Paclitaxel with CBQDs.

	Signals from CBQD (ppm)	Signals from Paclitaxel (ppm)	Signals from CBQD-Paclitaxel (ppm)
Disappeared	6.59 (s), 5.95 (s), 5.87 (s)	2.31 (s), 2.18 (s), 1.10 (s), and 0.80 (s)	N/A
Appeared new	N/A	N/A	6.94 (d), 6.31 (t), 5.83 (d) and 1.27 (t)

♦Correspond to lone Paclitaxel but were only visible in drug loading after hydrophilicity improved.

Table S8: ^{1}H-NMR (500 MHz, D_2O) summary of the binding of Paclitaxel with S-CBQDs.

	Signals from S-CBQD (ppm)	Signals from Paclitaxel (ppm)	Signals from SCBQD-Paclitaxel (ppm)
Disappeared	5.87 (s), 5.26 (s), 2.14 (s)	2.76 (s)	N/A
Appeared new	N/A	N/A	6.93 (s), 3.75 (s) and 3.60 (dd)

Table S9: ^{1}H-NMR (500 MHz, D_2O) summary of the binding of Paclitaxel with N-CBQDs.

	Signals from N-CBQD (ppm)	Signals from Paclitaxel (ppm)	Signals from NCBQD-Paclitaxel (ppm)
Disappeared	6.52 (s), 5.46 (dd), 5.31 (dd),1.85 (s)	2.18 (s)	N/A
Appeared new	N/A	N/A	6.90 (s), 6.29 (s), 5.81 (s), 3.71 (s)♦, 3.36 (s), 3.15 (s), 3.11(s), 1.98 (t)♦

♦Correspond to lone Paclitaxel but were only visible in drug loading after hydrophilicity improved.

Table S10: Partition coefficient results.

	Tetrandrine	Violacein	Paclitaxel
Lone drug	2.5[11]	3.34[21]	3.96[23]
CBQD	1.65 ± 0.16	1.17 ± 0.19	0.20 ± 0.06
S-CBQD	1.57 ± 0.32	1.73 ± 0.07	1.30 ± 0.19
N-CBQD	1.61 ± 0.05	2.22 ± 0.35	1.64 ± 0.05

Table S11: d-spacings of lone drugs, nanomaterials and drug-loaded nanomaterials.

Tetrandrine (T)	Violacein (V)	Paclitaxel (X)	CBQD	CBQD-T	CBQD-V	CBQD-X
6.6438	6.0053	10.3784	2.3626	3.1285	3.3808	2.1094
3.9642	2.9344	6.4557	1.9954	1.9277	2.1000	1.1600
3.6317	2.0178	5.7237	1.4277	1.0923	1.6983	
3.0057	1.5997	4.6669	1.2056	0.6389	1.2174	
2.1636		3.6809	0.9283	0.5488	1.0633	
		2.9198	0.8395		0.8053	
		2.2709	0.7931			
			0.7041			
			0.6392			
			0.5975			
			0.5619			

S-CBQD	SCBQD-T	SCBQD-V	SCBQD-X	N-CBQD	NCBQD-T	NCBQD-V	NCBQD-X
3.2453	1.8347	3.3171	3.3192	2.2145	2.7625	3.7659	3.1124
1.9725	1.1216	2.0552	2.0073	1.4416	1.6148	2.2073	1.8493
1.2339		1.7188	1.1654	1.1952	1.0620	1.2698	
		1.2197	0.9879	0.9155	0.8207		
		1.0462	0.7859	0.8369			
		0.8012	0.6777	0.7851			
			0.5934	0.6911			

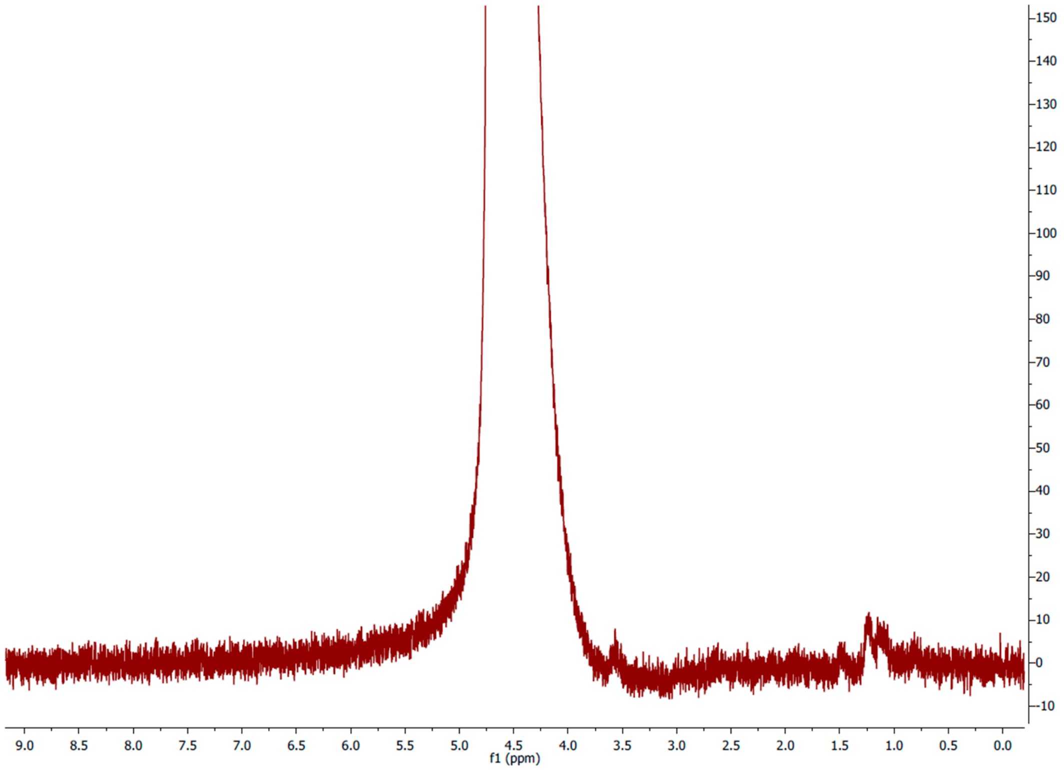

Figure S1: ^{1}H-NMR of Tetrandrine in D_2O.

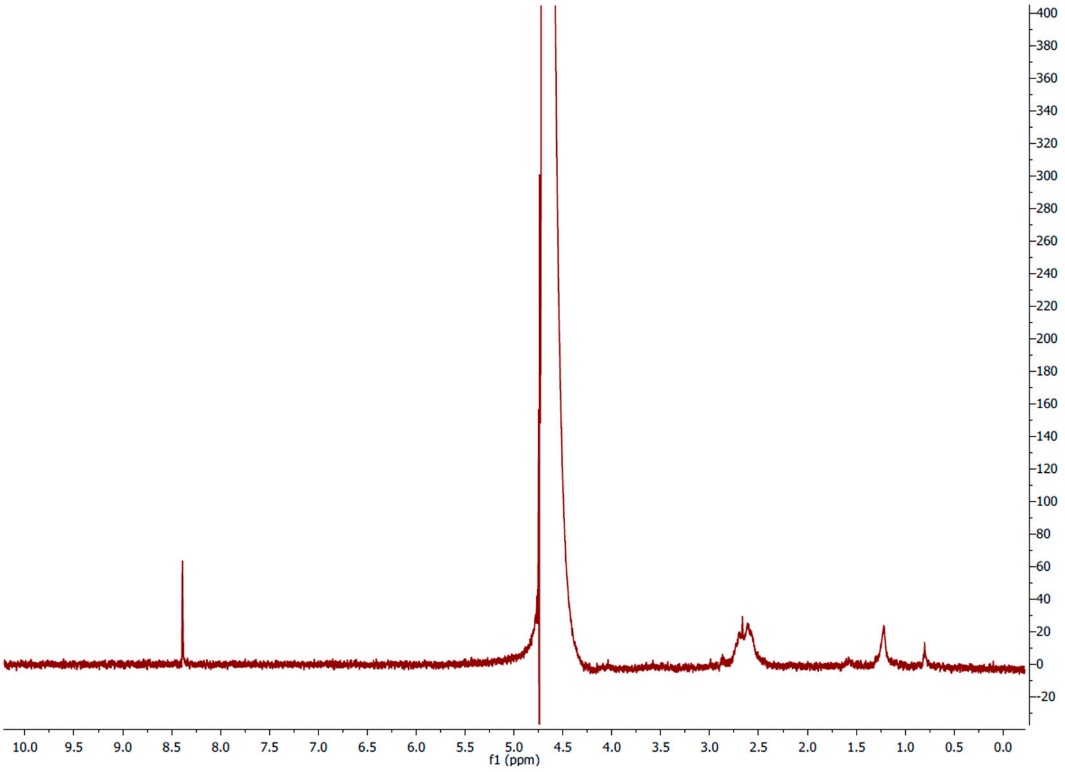

Figure S2: ^{1}H-NMR of Violacein in D_2O.

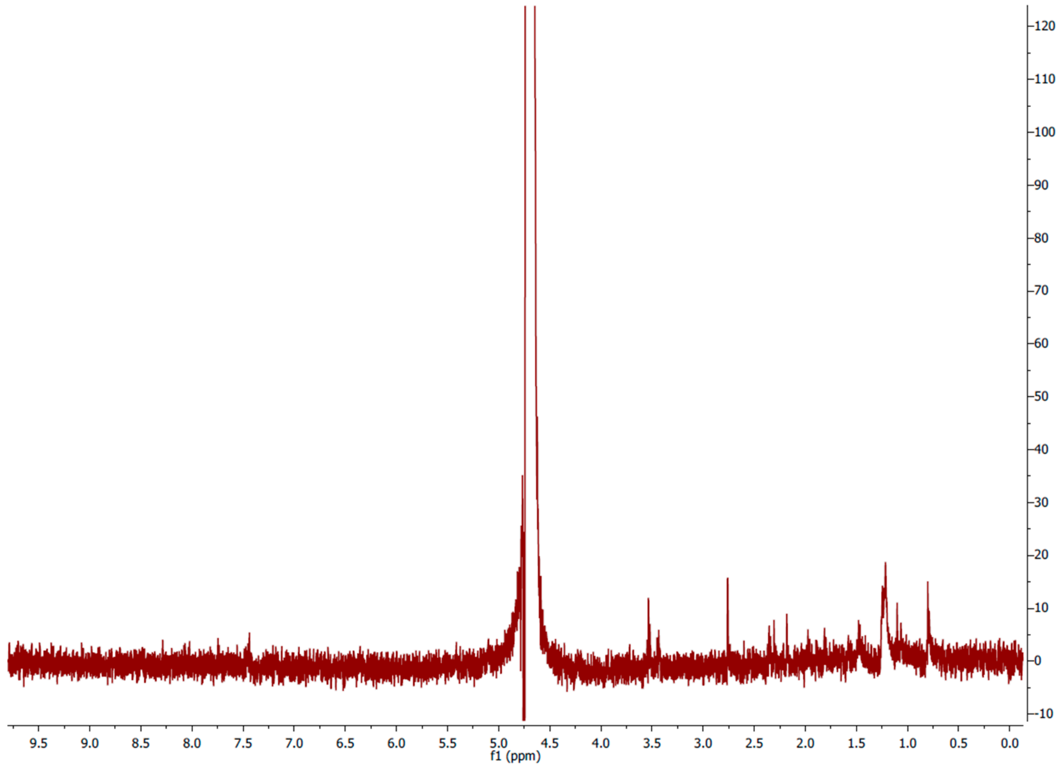

Figure S3: ^{1}H-NMR of Paclitaxel in D_2O.

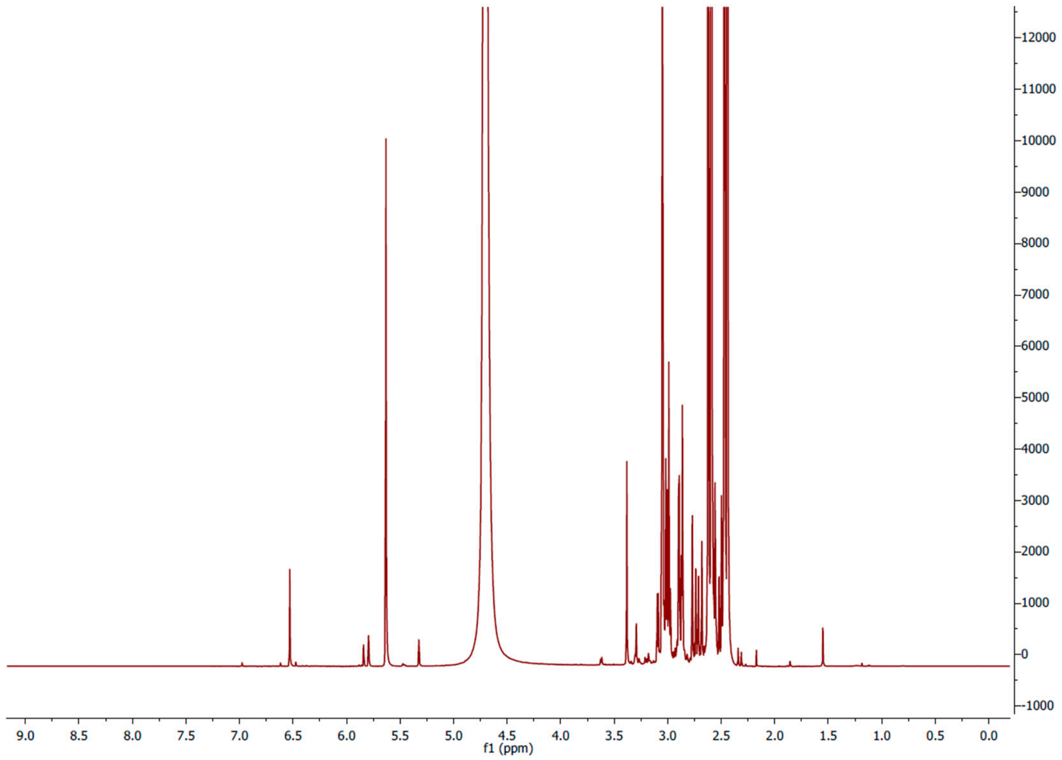

Figure S4: ^{1}H-NMR of CBQD in D_2O.

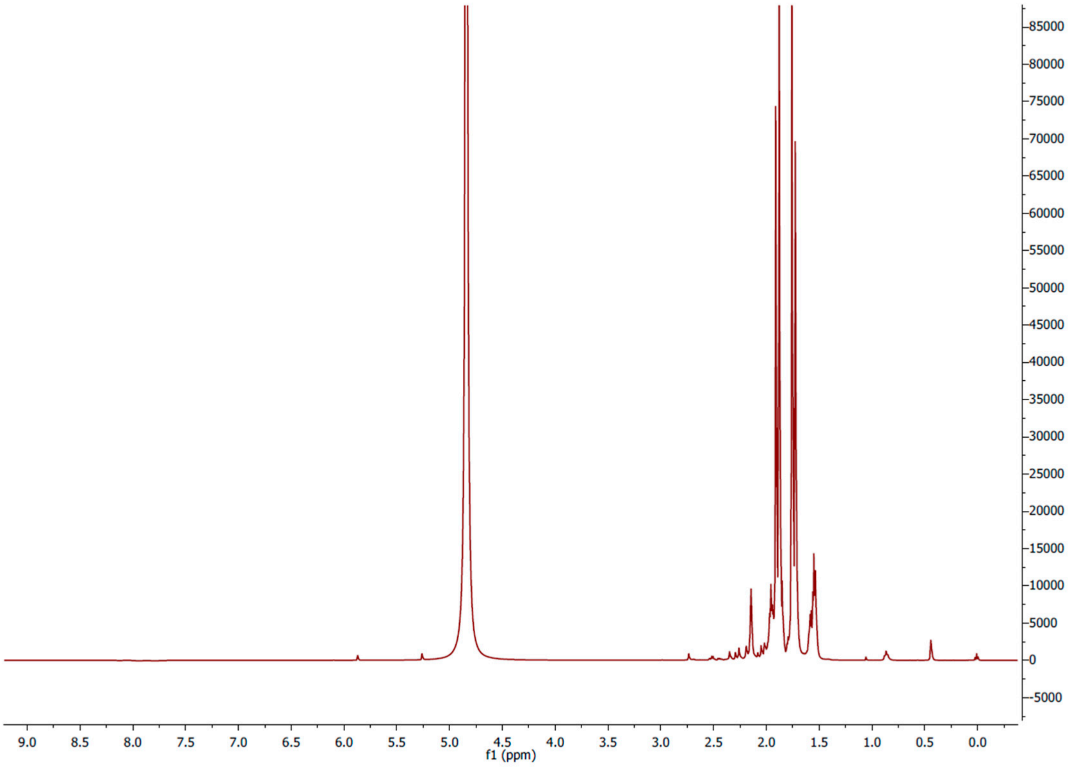

Figure S5: ^{1}H-NMR of S-CBQD in D_2O.

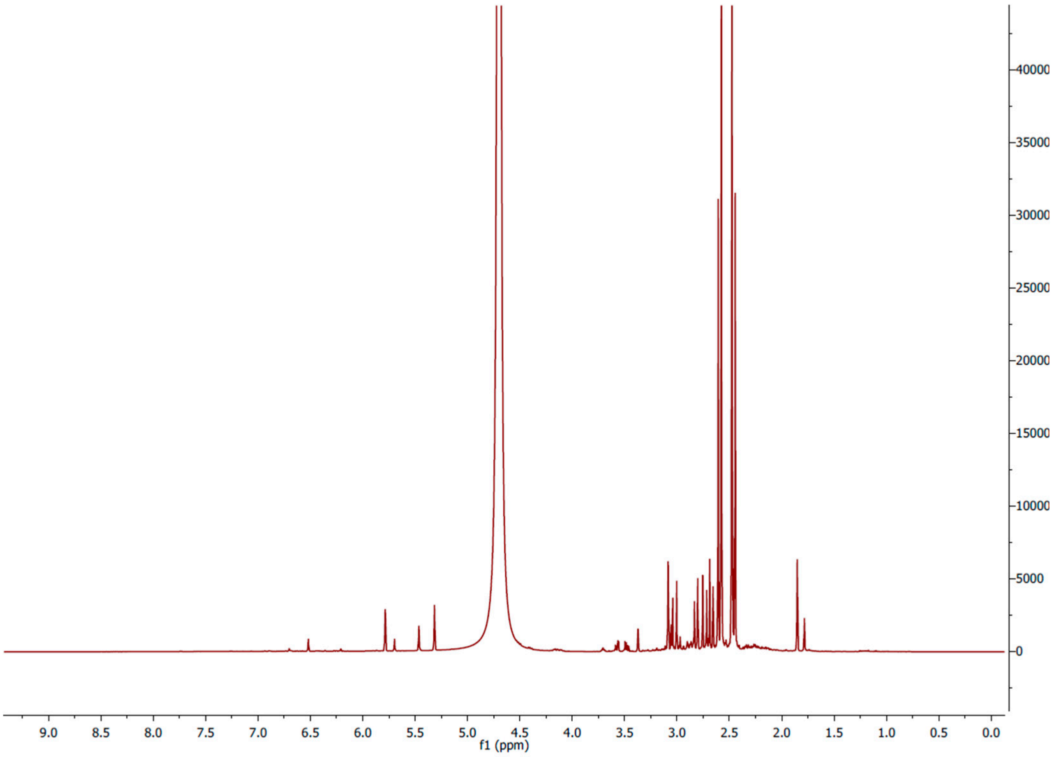

Figure S6: ^{1}H-NMR of N-CBQD in D_2O.

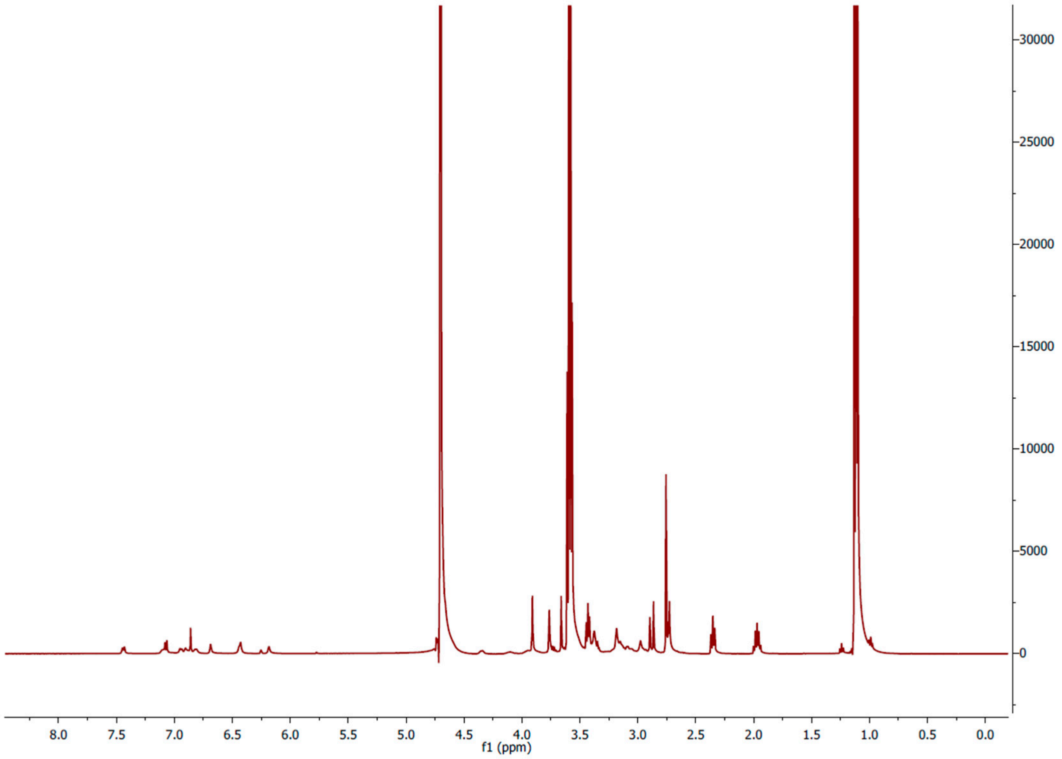

Figure S7: ^{1}H-NMR of CBQD-Tetrandrine in D_2O.

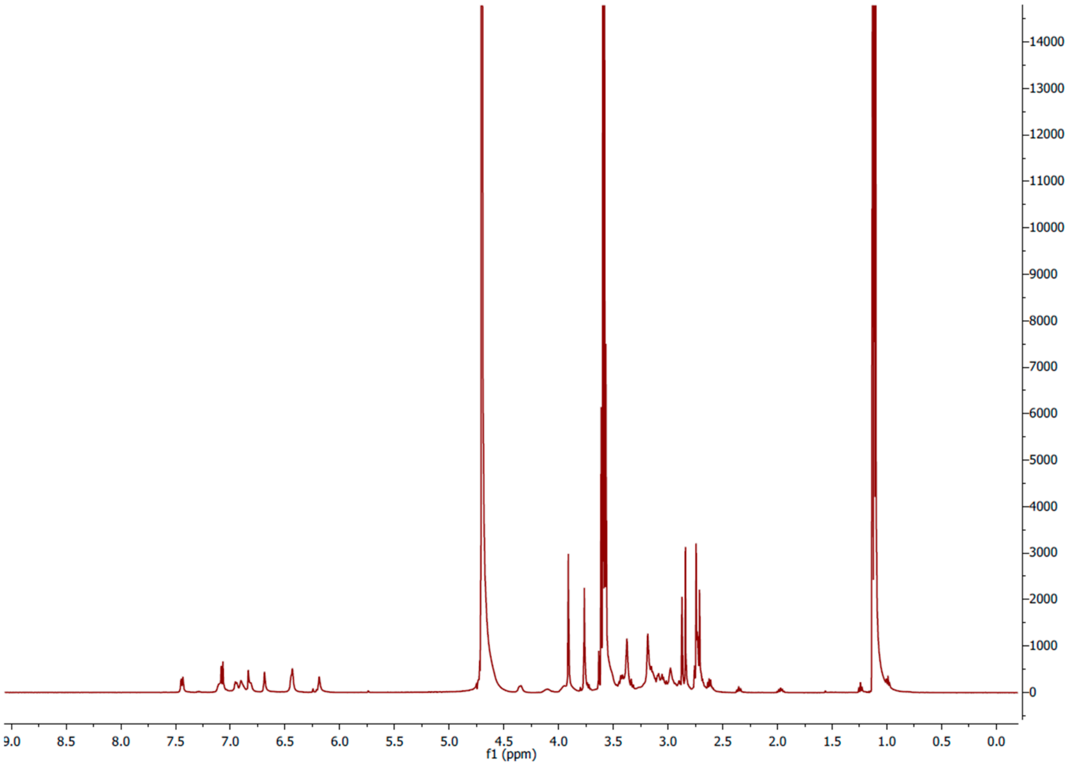

Figure S8: ^{1}H-NMR of SCBQD-Tetrandrine in D_2O.

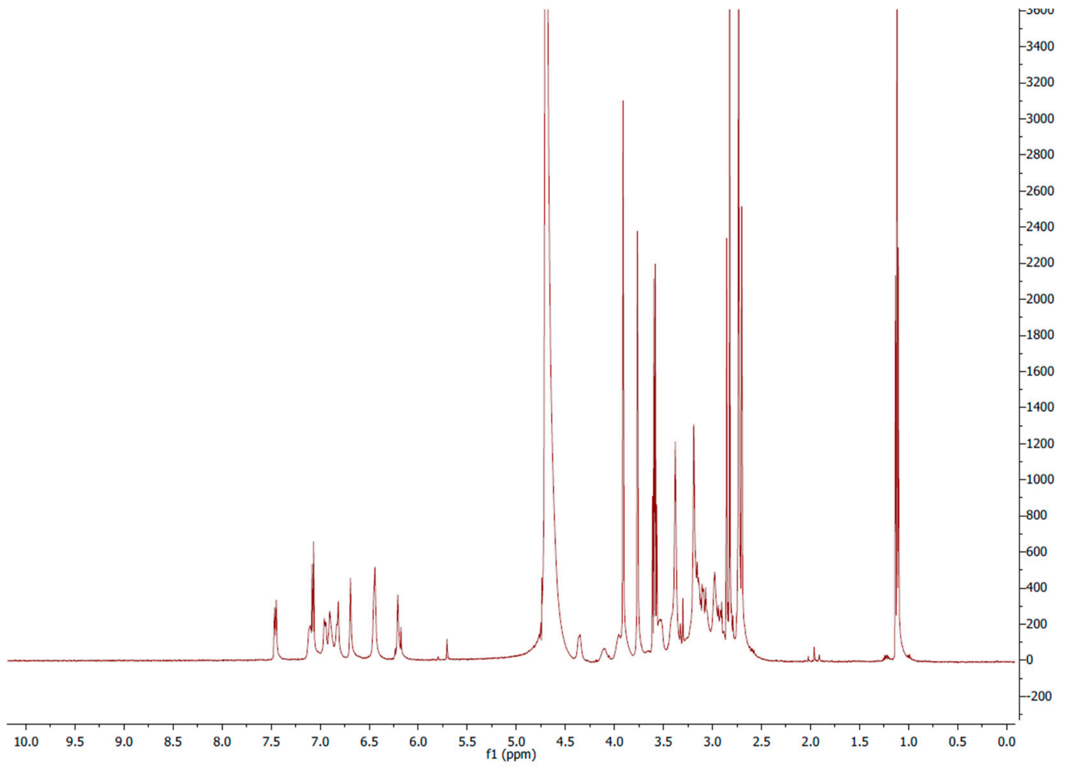

Figure S9: ^{1}H-NMR of NCBQD-Tetrandrine in D_2O.

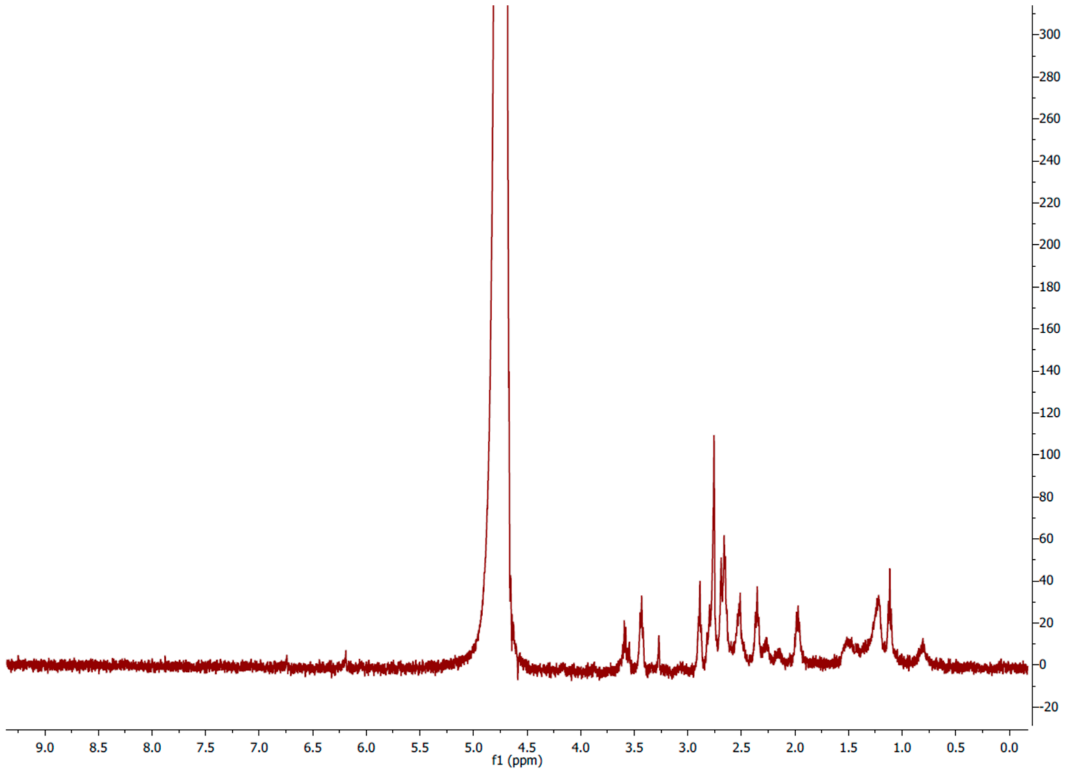

Figure S10: ^{1}H-NMR of CBQD-Violacein in D_2O.

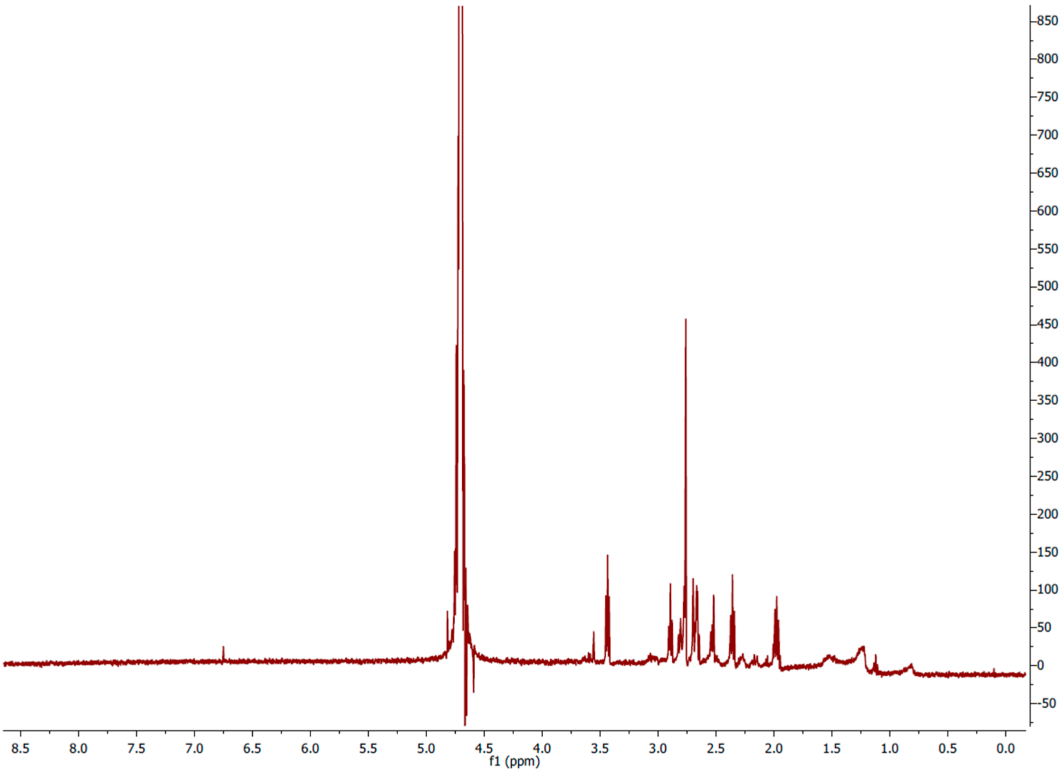

Figure S11: ^{1}H-NMR of SCBQD-Violacein in D_2O.

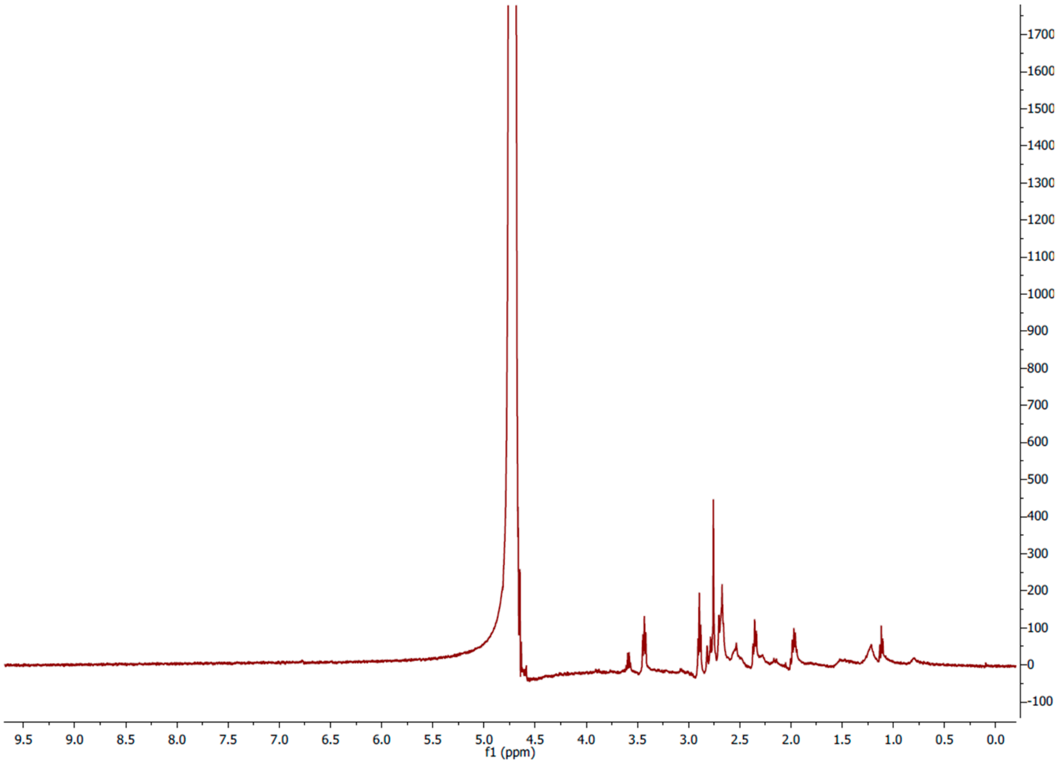

Figure S12: ^{1}H-NMR of NCBQD-Violacein in D_2O.

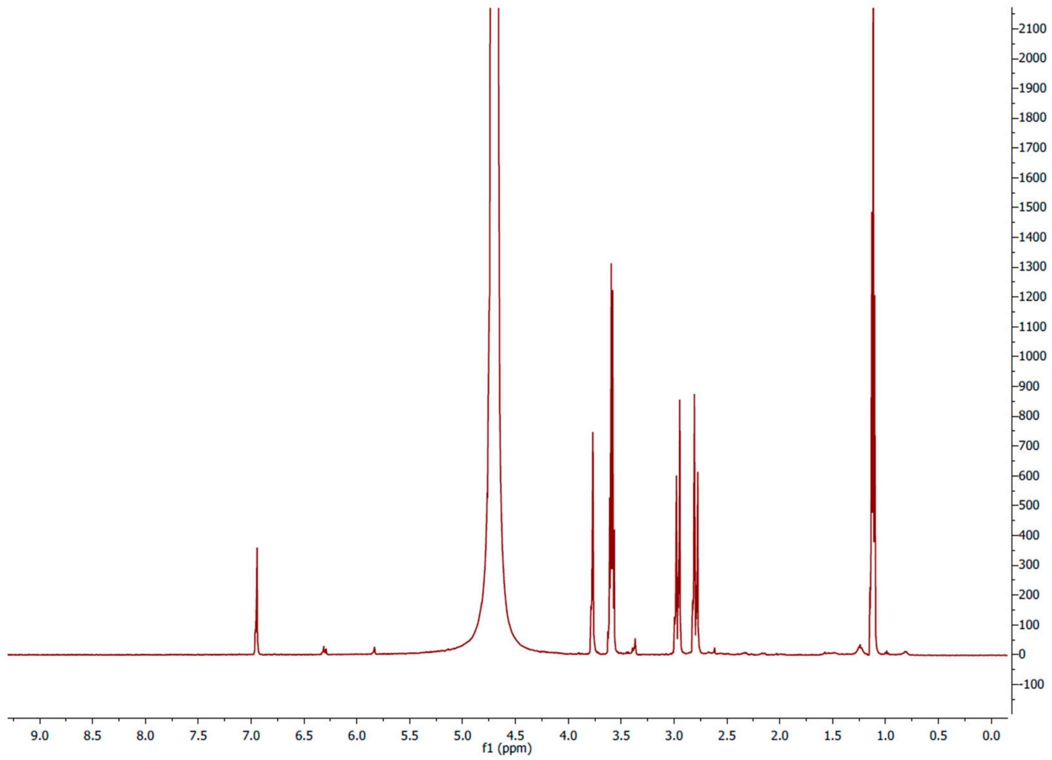

Figure S13: ^{1}H-NMR of CBQD-Paclitaxel in D_2O.

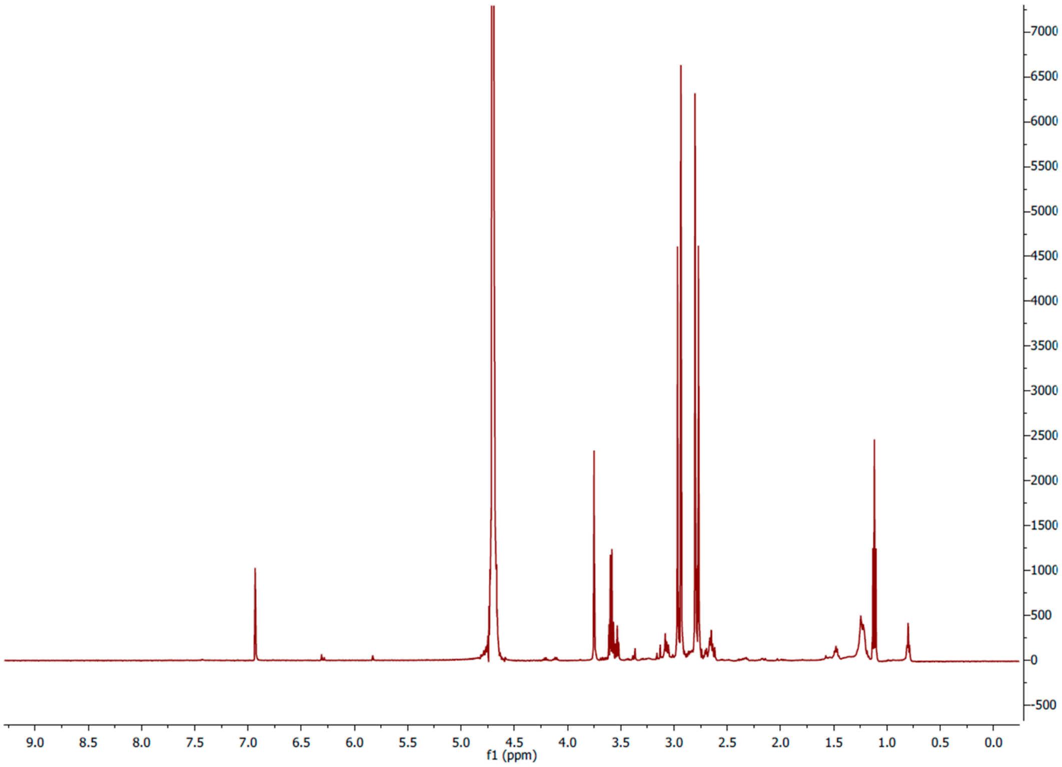

Figure S14: ^{1}H-NMR of SCBQD-Paclitaxel in D_2O.

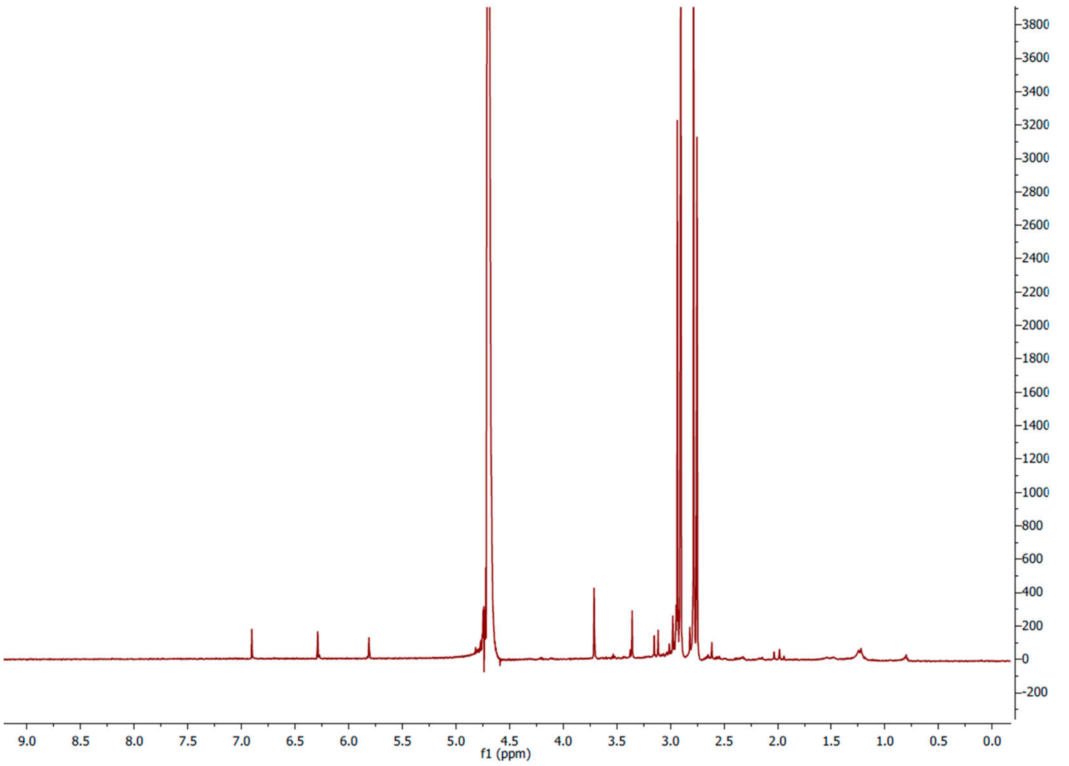

Figure S15: ^{1}H-NMR of NCBQD-Paclitaxel in D_2O.
